# MicroRNA-33 regulates sterol regulatory element-binding protein 1 expression in mice

**DOI:** 10.1038/ncomms3883

**Published:** 2013-12-03

**Authors:** Takahiro Horie, Tomohiro Nishino, Osamu Baba, Yasuhide Kuwabara, Tetsushi Nakao, Masataka Nishiga, Shunsuke Usami, Masayasu Izuhara, Naoya Sowa, Naoya Yahagi, Hitoshi Shimano, Shigenobu Matsumura, Kazuo Inoue, Hiroyuki Marusawa, Tomoyuki Nakamura, Koji Hasegawa, Noriaki Kume, Masayuki Yokode, Toru Kita, Takeshi Kimura, Koh Ono

**Affiliations:** 1 Department of Cardiovascular Medicine, Graduate School of Medicine, Kyoto University, Kyoto 606-8507, Japan; 2 Department of Clinical Innovative Medicine, Institute for Advancement of Clinical and Translational Science, Graduate School of Medicine, Kyoto University, Kyoto 606-8507, Japan; 3 Department of Internal Medicine (Endocrinology and Metabolism), Graduate School of Comprehensive Human Sciences, Nutrigenomics Research Group, Faculty of Medicine, and International Institute for Integrative Sleep Medicine (IIIS), World Premir International Research Center Initiative (WPI), University of Tsukuba, 1-1-1 Tennodai, Tsukuba, Ibaraki 305-8575, Japan; 4 Laboratory of Nutrition Chemistry, Division of Food Science and Biotechnology, Graduate School of Agriculture, Kyoto University, Kyoto 606-8502, Japan; 5 Department of Gastroenterology, Graduate School of Medicine, Kyoto University, Kyoto 606-8507, Japan; 6 Department of Pharmacology, Kansai Medical University, Moriguchi, Osaka 570-8506, Japan; 7 Division of Translational Research, National Hospital Organization, Kyoto Medical Center, Kyoto 612-8555, Japan; 8 Division of Clinical Pharmacy, Faculty of Pharmaceutical Sciences, Kobe Gakuin University, Kobe 650-8586, Japan; 9 Department of Cardiovascular Medicine, Kobe City Medical Center General Hospital, Kobe 650-0046, Japan; 10 These authors contributed equally to this work

## Abstract

MicroRNAs (miRs) are small non-protein-coding RNAs that bind to specific mRNAs and inhibit translation or promote mRNA degradation. Recent reports have indicated that miR-33, which is located within the intron of sterol regulatory element-binding protein (SREBP) 2, controls cholesterol homoeostasis and may be a potential therapeutic target for the treatment of atherosclerosis. Here we show that deletion of miR-33 results in marked worsening of high-fat diet-induced obesity and liver steatosis. Using miR-33^−/−^*Srebf1*^+/−^ mice, we demonstrate that SREBP-1 is a target of miR-33 and that the mechanisms leading to obesity and liver steatosis in miR-33^−/−^ mice involve enhanced expression of SREBP-1. These results elucidate a novel interaction between SREBP-1 and SREBP-2 mediated by miR-33 *in vivo*.

## Abstract (web summary)

The micro-RNA miR-33 is encoded by an intron of the gene encoding sterol regulatory-binding protein 2 (SREBP-2) and controls cholesterol homoeostasis. Here, Horie *et al.* identify SREBP-1 as a target of miR-33 and show that deletion of miR-33 promotes diet-induced obesity and liver steatosis in mice.

## Graphical abstract

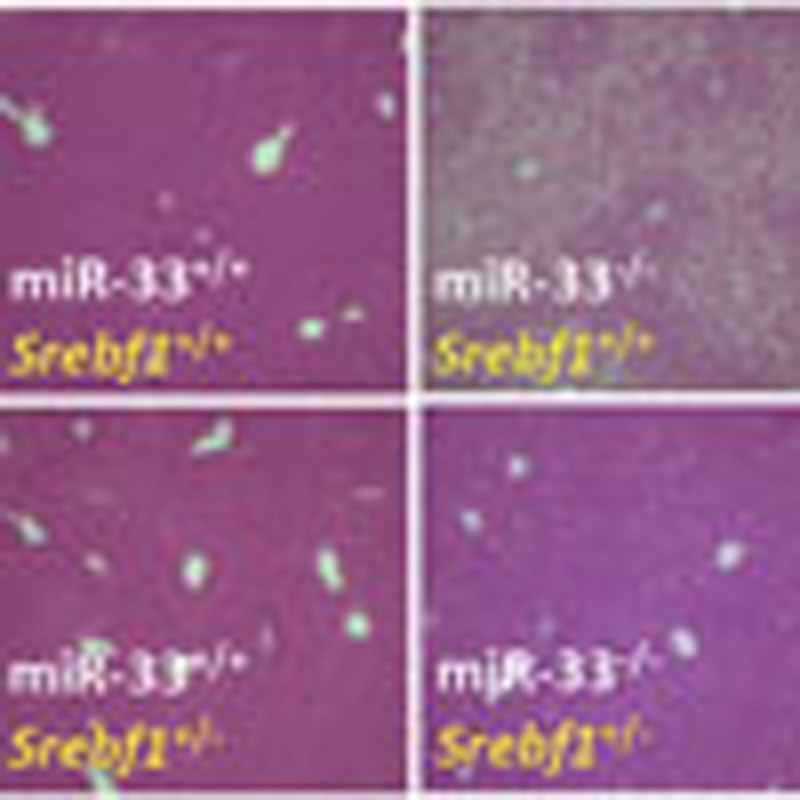

Sterol regulatory element-binding proteins (SREBPs) are the predominant transcription factors controlling the synthesis of cholesterol and fatty acids in the liver^1^. The family of SREBPs essentially encompasses two isoforms, SREBP-1 and SREBP-2, encoded by the corresponding genes *SREBF1* and *SREBF2* (refs 2, 3). In contrast to SREBP-2, SREBP-1 is transcribed into two major splicing variants, SREBP-1a and SREBP-1c, which differ only in their first exon through the use of alternative promoters^2^,^3^. Although there is some functional overlap among the three SREBP isoforms^4^, these proteins regulate different metabolic pathways. SREBP-2 is the master regulator of cholesterol synthesis and metabolism, whereas SREBP-1c controls fatty acid synthesis in the liver and adipose tissue^5^. In replicating tumour cell lines, SREBP-1a mostly transactivates both lipogenic and cholesterogenic genes. Although SREBP-1a and SREBP-1c share the same bHLH and regulatory domains, SREBP-1a is a stronger activator than SREBP-1c owing to a longer amino-terminal transactivation domain^6^. Therefore, SREBP-1a, -1c and -2 have specific roles in the regulation of cholesterol and fatty acids. In order to fine-tune cellular metabolism efficiently, it may be important to regulate their functions in an interdependent manner. However, limited evidence has been obtained about the potential interactions between SREBP-1 and SREBP-2 until date.

MicroRNAs (miRs) are small non-protein-coding RNAs that bind to specific mRNAs and inhibit translation or promote mRNA degradation. miR-33 is encoded in an intron of *SREBF2*. The sequence of miR-33 is identical, and the stem-loop of the pre-miRNA is highly conserved in mammals^7^,^8^,^9^,^10^. Recent reports, including ours, have indicated that miR-33 controls ABCA1 expression and reduces HDL-C levels, and that miR-33 is a potential target for the treatment of atherosclerosis^11^,^12^. To determine the organ/cell type-specific function of miRs in the long term *in vivo*, studies on miRNA-deficient mice and analysis of specific organ/cell types from these mice are needed. Therefore, we generated miR-33-deficient mice and studied their phenotypes. We noted that miR-33-deficient mice gradually gained more weight than control mice, and the obese phenotype was evident after 26 weeks of age when receiving normal chow (NC). When we fed them a high-fat diet (HFD), miR-33-deficient mice became severely obese and suffered from liver steatosis. Microarray analysis showed that genes involved in fatty acid metabolism were upregulated in miR-33^−/−^ mice fed NC before becoming obese. We searched for potential target genes of miR-33 in a public database (TargetScan, http://www.targetscan.org), and found that one of the targets of miR-33 is SREBP-1. *In vitro* experiments indicated that SREBP-1 is a likely target of miR-33. We further intercrossed miR-33^−/−^ mice with *Srebf1*^+/−^ mice and fed them HFD. The difference in body weight (BW) between miR-33^−/−^*Srebf1*^+/+^ mice and miR-33^−/−^*Srebf1*^+/−^ mice decreased and hepatic steatosis was reversed in miR-33^−/−^*Srebf1*^+/−^ mice compared with miR-33^−/−^*Srebf1*^+/+^ mice under pair-feeding conditions. These data demonstrate that miR-33 targets SREBP-1 *in vivo*.

In the present study, we demonstrated that miR-33 deficiency increases SREBP-1 levels, fatty acid synthesis, and fatty acid accumulation in the liver and adipose tissue. These results indicate a novel relationship between SREBP-1 and SREBP-2 through miR-33.

## Results

### miR-33-KO mice become obese and develop hepatic steatosis

**Figure 1:**
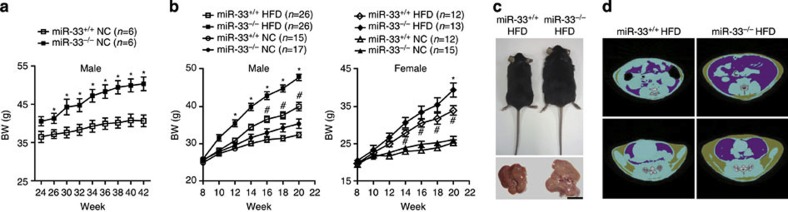
miR-33^−/−^ mice become obese and develop hepatic steatosis. (**a**) Development of BW of miR-33^+/+^ and miR-33^−/−^ male mice fed NC. *P<0.05 versus NC-fed miR-33^+/+^ mice. Statistical comparisons were made by Student’s *t*-test. (**b**) Development of BW of miR-33^+/+^ and miR-33^−/−^ mice fed or not fed HFD. *P<0.05 versus HFD-fed miR-33^+/+^ mice, #*P*<0.05 versus NC-fed miR-33^+/+^ mice. Statistical comparisons were made by one-way analysis of valiance test. (**c**) Representative image of miR-33^+/+^ and miR-33^−/−^ mice fed with a HFD. Lower images show the livers of these mice. Scale bars, 1.0 cm. (**d**) Representative CT images of miR-33^+/+^ and miR-33^−/−^ mice fed HFD. Values are the means±s.e.m.

**Figure 2:**
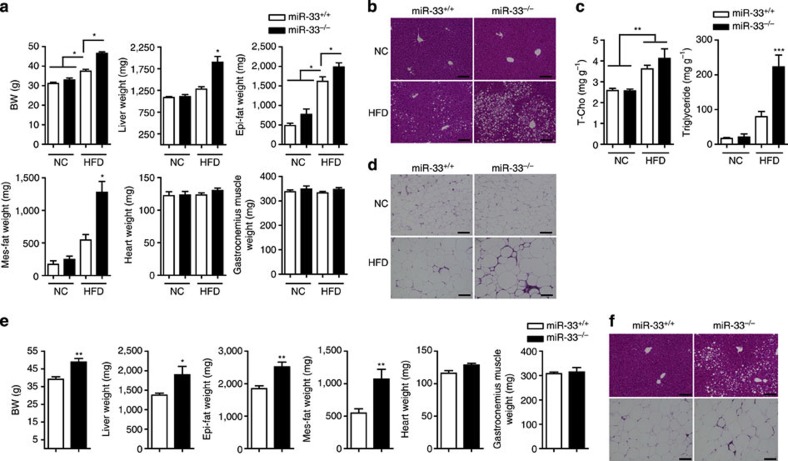
Pathophysiological features of miR-33^−/−^ mice. (**a**) Body, liver, adipose tissue (epi-fat; epididymal fat, mes-fat; mesenteric fat), heart and muscle weights of miR-33^+/+^ and miR-33^−/−^ mice fed or not fed HFD (*n*=12–13 for NC, *n*=21 for HFD each. *P<0.05 in one-way analysis of valiance test). (**b**) Representative microscopic images of the livers of miR-33^+/+^ and miR-33^−/−^ mice fed or not fed HFD. Scale bars, 200 μm. (**c**) Cholesterol and triglyceride levels in the livers of miR-33^+/+^ and miR-33^−/−^ mice fed or not fed HFD (*n*=5 each. **P<0.01, ***P<0.001 in one-way analysis of valiance test). (**d**) Representative microscopic images of the adipose tissue (epididymal fat) of miR-33^+/+^ and miR-33^−/−^ mice fed or not fed HFD. Scale bars, 200 μm. (**e**) Body, liver, adipose tissue, heart and muscle weights of miR-33^+/+^ and miR-33^−/−^ mice fed NC at the age of 50 weeks (*n*=6 each, *P<0.05, **P<0.01 in Student’s *t*-test). (**f**) Representative microscopic images of the liver and the adipose tissue of miR-33^+/+^ and miR-33^−/−^ mice fed NC at the age of 50 weeks. Scale bars, 200 μm. Values are the means±s.e.m.

Twenty-six-week-old male miR-33-knockout mice weighed more than wild-type (WT) littermates after being fed NC (Fig. 1a). Up to 24 weeks of age, the BWs of the miR-33^−/−^ mice and those of age- and sex-matched miR-33^+/+^ control mice did not differ, but 26-week-old male miR-33^−/−^ mice were 20% heavier than controls. After feeding with HFD from 8 to 20 weeks of age, miR-33^−/−^ mice become markedly obese compared with controls of both genders (Fig. 1b,c). Computed tomography (CT) of 20-week-old miR-33^+/+^ and miR-33^−/−^ mice fed HFD showed a severe increase in body fat of miR-33^−/−^ mice compared with miR-33^+/+^ mice (Fig. 1d). We estimated fat weight from CT values because there is a good correlation of visceral fat weight and calculated weight from CT values (Supplementary Fig. S1a). Both visceral and subcutaneous fat weights were higher in miR-33^−/−^ mice fed HFD (Supplementary Fig. S1b). Figure 2a indicates that the increased BW was caused by an increase in liver and adipose tissue weight. The livers of miR-33^−/−^ mice fed HFD were severely enlarged and pale in colour (Fig. 1c). Histological examination revealed that miR-33^−/−^ mice fed HFD developed severe fatty liver with the accumulation of lipid droplets (Fig. 2b). We measured total cholesterol and triglyceride levels in the liver and found that triglyceride levels were significantly increased in the liver of miR-33^−/−^ mice fed HFD compared with miR-33^+/+^ mice fed HFD and mice fed NC (Fig. 2c right). On the other hand, cholesterol levels in the liver were increased in mice fed HFD compared with mice fed NC and there was no difference between miR-33^+/+^ and miR-33^−/−^ mice (Fig. 2c left). Figure 2d shows the increase in adipocyte size with the accumulation of infiltrated cells in white adipose tissue in miR-33^−/−^ mice fed HFD. It is of note that the same phenotypes as those of miR-33^−/−^ mice fed HFD were also observed in miR-33^−/−^ mice fed NC at 50 weeks of age (Fig. 2e,f). Thus, genetic ablation of miR-33 induces obesity and hepatic steatosis.

### miR-33-KO mice have abnormal glucose and insulin tolerance

**Figure 3:**
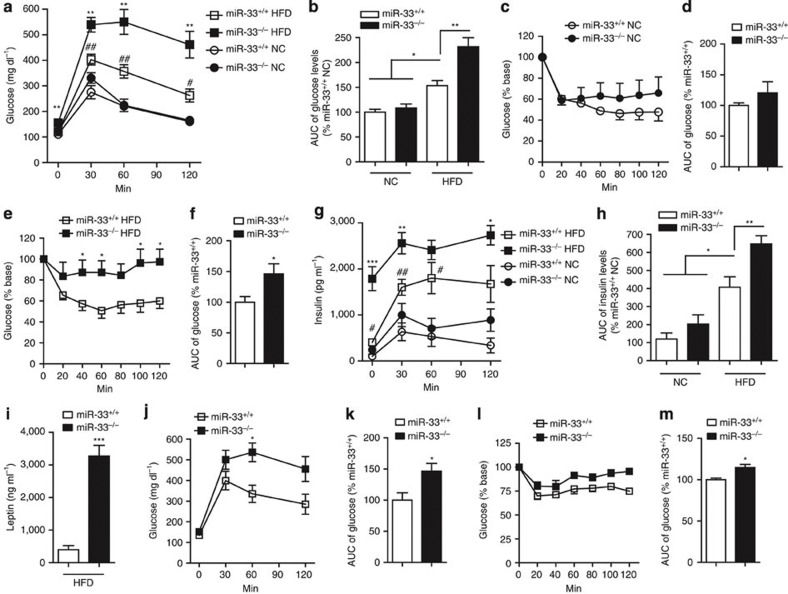
Analysis of glucose and insulin tolerance. (**a**,**b**) Serial changes in glucose levels (**a**) and area under curve (AUC) of glucose levels (**b**) after intraperitoneal injection of glucose in miR-33^+/+^ and miR-33^−/−^ mice fed or not fed HFD (*n*=6 for NC, *n*=11–12 for HFD each. *P<0.05 versus mice fed NC. **P<0.01 versus miR-33^+/+^ mice fed HFD. ^#^*P*<0.05 versus miR-33^+/+^ mice fed NC, ^##^*P*<0.01 versus miR-33^+/+^ mice fed NC in one-way analysis of valiance test). (**c**,**d**) Serial changes in glucose levels (**c**) and AUC of glucose levels (**d**) after intraperitoneal injection of insulin in miR-33^+/+^ and miR-33^−/−^ mice fed NC (*n*=5 each). (**e**,**f**) Serial changes in glucose levels (**e**) and AUC of glucose levels (**f**) after intraperitoneal injection of insulin in miR-33^+/+^ and miR-33^−/−^ mice fed HFD (*n*=9 each, *P<0.05 in Student’s *t*-test). (**g**) Serial changes in insulin levels after intraperitoneal injection of glucose in miR-33^+/+^ and miR-33^−/−^ mice fed or not fed HFD (*n*=6 for NC, *n*=11–12 for HFD each. *P<0.05 versus miR-33^+/+^ mice fed HFD, **P<0.01 versus miR-33^+/+^ mice fed HFD, ***P<0.001 versus miR-33^+/+^ mice fed HFD, ^#^*P*<0.05 versus miR-33^+/+^ mice fed NC, ^##^*P*<0.01 versus miR-33^+/+^ mice fed NC in one-way analysis of valiance test). (**h**) AUC of insulin levels after intraperitoneal injection of glucose in miR-33^+/+^ and miR-33^−/−^ mice fed or not fed HFD (*n*=6 for NC, *n*=11–12 for HFD each, *P<0.05, **P<0.01 in one-way analysis of valiance test). (**i**) Serum leptin levels in miR-33^+/+^ and miR-33^−/−^ mice fed HFD (*n*=10 for each, ***P<0.001 in Student’s *t*-test). (**j**,**k**) Serial changes in glucose levels (**j**) and AUC of glucose levels (**k**) after intraperitoneal injection of glucose in miR-33^+/+^ and miR-33^−/−^ mice fed NC at the age of 50 weeks (*n*=6 each, *P<0.05 in Student’s *t*-test). (**l**,**m**) Serial changes in glucose levels (**l**) and AUC of glucose levels (**m**) after intraperitoneal injection of insulin in miR-33^+/+^ and miR-33^−/−^ mice fed NC at the age of 50 weeks (*n*=6 each, *P<0.05 in Student’s *t*-test). Values are the means±s.e.m.

miR-33^−/−^ mice fed HFD from 8 weeks to 20 weeks of age showed higher fasting glucose levels and severely impaired glucose tolerance at 20 weeks (Fig. 3a,b).

However, miR-33^−/−^ mice fed NC showed the same glucose levels as miR-33^+/+^ mice at this age. Baseline glucose levels of NC-fed miR-33^+/+^ mice, NC-fed miR-33^−/−^ mice, HFD-fed miR-33^+/+^ mice and HFD-fed miR-33^−/−^ mice were 110.5±8.3, 122±2.5, 120.5±5.6 and 155.6±6.7 mg dl^−1^, respectively (All values represent mean±s.e.m.). Impaired insulin tolerance was observed only in miR-33^−/−^ mice fed HFD (Fig. 3c–f). Insulin levels in intraperitoneal glucose tolerance test (IPGTT) were significantly elevated in miR-33^−/−^ mice fed HFD (Fig. 3g,h). Plasma leptin levels were also elevated in miR-33^−/−^ mice fed HFD (Fig. 3i). Impaired glucose tolerance and insulin tolerance were also evident at the age of 50 weeks even in mice fed NC (Fig. 3j–m).

Serum levels of ALP, T-cho and HDL-C were elevated in miR-33^−/−^ mice compared with that in WT mice at the age of 20 weeks, as indicated in our previous report (Table 1)^10^. When these mice were fed HFD from 8 to 20 weeks of age, increases in serum levels of AST, ALT, NEFA and LDL-C became evident (Table 1). Similar elevation of T-cho was observed in miR-33^−/−^ mice fed NC at the age of 50 weeks compared with controls (Supplementary Table S2).

### miR-33-KO mice find HFD more palatable

**Figure 4:**
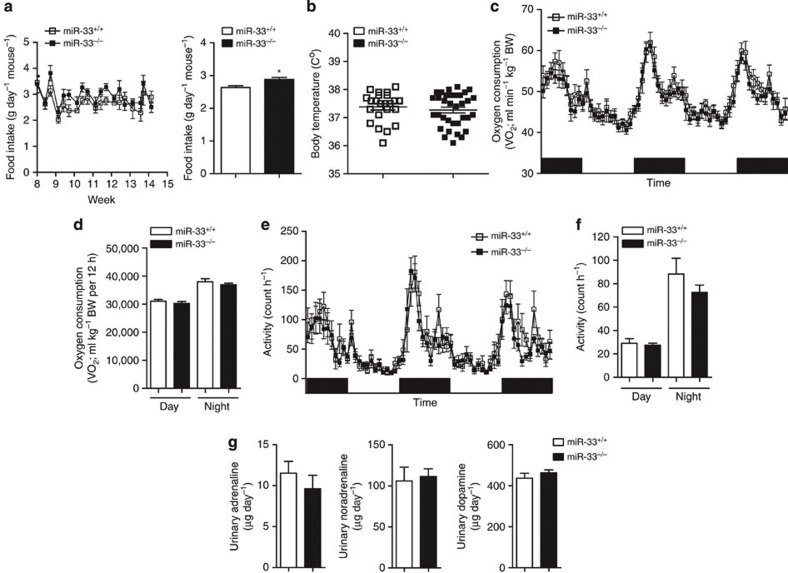
Analysis of energy balance. (**a**) Serial changes in food intake of miR-33^+/+^ and miR-33^−/−^ mice fed HFD in metabolic cages (*n*=5–6 each, *P<0.05 in Student’s *t*-test). (**b**) Body temperature of miR-33^+/+^ and miR-33^−/−^ mice at 16 weeks of age (*n*=23, 34 each). (**c**) Oxygen consumption rate of miR-33^+/+^ and miR-33^−/−^ mice fed NC at 16 weeks of age (*n*=8 each). (**d**) Oxygen consumption during 12 h by miR-33^+/+^ and miR-33^−/−^ mice fed NC at 16 weeks of age (*n*=8 each). (**e**) Serial changes in activity of miR-33^+/+^ and miR-33^−/−^ mice fed NC at 16 weeks of age (*n*=8 each). (**f**) Day and night activity of miR-33^+/+^ and miR-33^−/−^ mice fed NC at 16 weeks of age (*n*=8 each). (**g**) Urinary secretion of adrenaline, noradrenaline and dopamine (*n*=3–4 each). Values are the means±s.e.m.

Food intake, as analysed by housing in metabolic cages, was higher in miR-33^−/−^ mice fed HFD than that in their control counterparts (Fig. 4a). The difference in food intake was only observed when they were fed with HFD (Supplementary Fig. S2a, b), which suggests that miR-33^−/−^ mice find HFD more palatable. These mice showed similar body temperatures (37.38 °C versus 37.27 °C) and O_2_ consumption rate or activity did not differ between these strains during the day or night at the age of 16 weeks when fed NC (Fig. 4c–f). Moreover, urinary excretion of adrenaline, noradrenaline and dopamine were also the same between these strains at the same age (Fig. 4g).

### miR-33 regulates SREBP-1 expression *in vivo*

**Figure 5:**
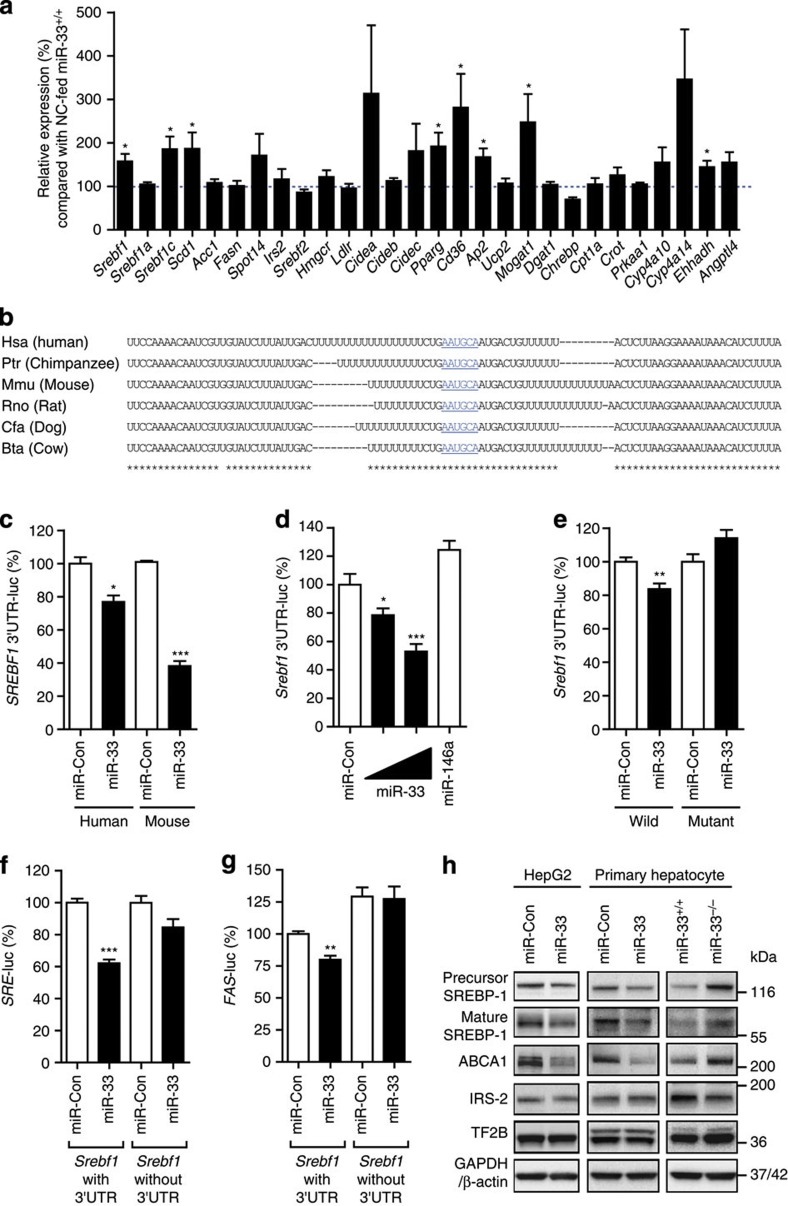
*Srebf1* is a miR-33 target gene. (**a**) Relative changes in lipid metabolism-related genes in the livers of miR-33^−/−^ mice compared with miR-33^+/+^ mice fed NC at 16 weeks of age. (*n*=5–8 each,*P<0.05 in Student’s *t*-test). (**b**) Conservation of miR-33 target regions in the 3′UTR of *Srebf1*. Underlined sequences are the potential binding site of miR-33 seed sequences. * indicates the conservation among spieces. (**c**) 3′UTR reporter assay used to verify the target. Luciferase reporter activity of human and mouse SREBP-1 gene 3′UTR constructs in 293T cells overexpressing miR-control (miR-Con) and miR-33 (*n*=4 each, *P<0.05 and ***P<0.001 in Student’s *t*-test). (**d**) miR-33 dose-dependent changes in luciferase reporter activity of mouse *Srebf1* 3′UTR construct in 293T cells. miR-Con and miR-146a is used as a negative control (*n*=4 each, *P<0.05 and ***P<0.001 in one-way analysis of valiance test). (**e**) Luciferase reporter activity of the WT or mutant *Srebf1* 3′UTR at the potential miR-33 binding site in 293T cells (*n*=4 each, **P<0.01 in Student’s *t*-test). (**f**,**g**) Luciferase reporter activity of *SRE*-promoter (**f**) or *FAS*-promoter (**g**) in 293T cells. 293T cells were co-transfected with mouse *Srebf1* with the full-length 3′UTR or without the 3′UTR, along with expression plasmids for miR-negative control, or miR-33. Values are the mean±s.e. (*n*=4 each, **P<0.01 versus miR-Con. ***P<0.001 versus miR-Con in one-way analysis of valiance test). (**h**) Western blotting analysis of SREBP-1, ABCA1, and IRS-2 in miR-33 transduced HepG2 cells and primary hepatocytes and hepatocytes prepared from miR-33^+/+^ and miR-33^−/−^ mice. Representative western blot images are shown (*n*=4). Values are the means±s.e.m.

In order to determine the cause of the phenotypic changes observed in miR-33^−/−^mice fed HFD or in older miR-33^−/−^ mice, we analysed the gene expression profiles by microarray analysis using the livers of miR-33^+/+^ and miR-33^−/−^ mice fed NC at the age of 16 weeks when their weights were the same. The pathways altered in the livers of miR-33^−/−^ mice were determined by GenMAPP analysis ( http://www.genmapp.org/about.html). Most strikingly, the fatty acid metabolism pathway showed the highest Z score (Supplementary Table S3). We picked up genes related to fatty acid metabolism and validated their expression levels in the liver by quantitative RT–PCR (PCR with reverse transcription). Interestingly, significant differences were observed in the expression levels of several lipogenic genes including *Srebf1, Pparg* and its downstream genes (Fig. 5a). We also measured *de novo* hepatic fatty acid synthesis rate, as previously described^13^,^14^. It was increased significantly in the miR-33^−/−^ mice compared with that of the miR-33^+/+^ mice (Supplementary Fig. S3a). The *Srebf1* 3′UTR has a potential binding site for miR-33 in many species (TargetScan; http://www.targetscan.org; Fig. 5b). Overexpression of miR-33 reduced the luciferase activity of a reporter gene fused with *Srebf1* 3′UTR sequences from humans and mice (Fig. 5c). Moreover, miR-33 decreased luciferase activity dose-dependently, whereas miR-146a, which has no binding site in the *Srebf1* 3′UTR, could not (Fig. 5d). Mutation in this binding site abolished the reduction of luciferase activity in 293T cells (Fig. 5e). The same results were also obtained in COS-7 cells (Supplementary Fig. S3b, c). We also measured the activity of SREBP-1 by sterol regulatory element (*SRE*) and fatty acid synthase (*FAS*) promoter reporter analysis by the use of *Srebf1* with or without the 3′UTR. Luciferase activity of the *SRE* and *FAS* reporter genes was significantly reduced by miR-33 expression when *Srebf1* with the 3′UTR was present. This reduction was not observed in the experiments conducted with *Srebf1* without the 3′UTR (Fig. 5f,g). Overexpression of miR-33 reduced protein levels of SREBP-1 and ABCA1 but not of IRS-2 in HepG2 cells (Fig. 5h and Supplementary Fig. S4a). The decrease in *SREBF1* expression was mainly caused by reduction in *SREBF1*c (Supplementary Fig. S4b). Overexpression of miR-33 also reduced the protein levels of SREBP-1 and ABCA1 but not of IRS-2 in miR-33^+/+^ primary hepatocytes (Fig. 5h and Supplementary Fig. S4c). It was confirmed that miR-33^−/−^ mice had higher protein expression levels of SREBP-1 and ABCA1 but not of IRS-2 (Fig. 5h and Supplementary Fig. S4d). We measured the expression levels of lipogenic genes in the primary hepatocyte transduced with miR-33 or the control. As shown in Supplementary Fig. S4e, expression levels of *Srebf1*, *Abca1* and several lipogenic genes were downregulated. Moreover, *Srebf1*, *Abca1* and several lipogenic genes were upregulated in miR-33^−/−^ primary hepatocytes compared with miR-33^+/+^ primary hepatocytes (Supplementary Fig. S4f). SREBP-1 levels were further enhanced in miR-33^−/−^ mice fed HFD (Supplementary Fig. S5a). We also measured the levels of AMPKα, previously described as a potential miR-33 target, but we could not detect any difference between miR-33^+/+^ and miR-33^−/−^ mice (Supplementary Fig. S5b). We further checked whether PPAR-γ is regulated by miR-33 in primary hepatocytes. Overexpression of miR-33 did not change the protein expression level of PPAR-γ after transduction in HepG2 cells (Supplementary Fig. S5c) and primary hepatocytes (Supplementary Fig. S5d), and found non-enhancement in miR-33^−/−^ mice (Supplementary Fig. S5d). Moreover, we conducted peroxisome proliferator-activated receptor response elements (*PPRE*)-driven luciferase assay in HepG2 cells. No difference was observed in *PPRE* activity in control and miR-33 transduced cells with or without PPAR ligand pioglitazone (Supplementary Fig. S5e). Therefore, these results indicate that *Pparg* is not a direct target of miR-33.

### SREBP-1 is regulated by endogenous changes in miR-33 *in vitro*

**Figure 6:**
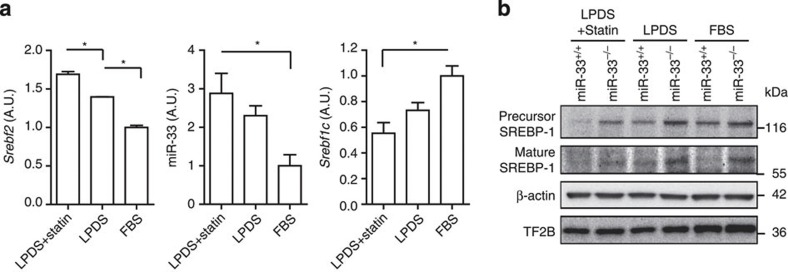
SREBP-1 is regulated by endogenous changes in miR-33 *in vitro*. (**a**) RNA expression levels in *Srebf2*, miR-33 and *Srebf1c* in primary hepatocytes cultured in DMEM supplemented with 5% FBS or 5% LPDS with or without statin treatment. Values are the mean±s.e.m. (*n*=3 each for *Srebf2* and *Srebf1c*, *n*=4–6 each for miR-33, *P<0.05 in one-way analysis of valiance test). (**b**) Protein levels of SREBP-1 in primary hepatocytes cultured in DMEM supplemented with 5% FBS or 5% LPDS with or without statin treatment. Representative western blot images are shown (*n*=4).

We further attempted to confirm whether the expression of SREBP-1 was affected by endogenous changes in miR-33 expression via modulating the cellular cholesterol level in primary hepatocytes. When the cells were depleted of sterols by prior incubation in medium containing lipoprotein-deficient serum (LPDS) with or without pitavastatin, mRNA levels of *Srebf2* and miR-33 were significantly increased in parallel (Fig. 6a). In this situation, *Srebf1c* and protein levels of SREBP-1 were decreased in miR-33^+/+^ primary hepatocytes, whereas they were still elevated in miR-33^−/−^ hepatocytes (Fig. 6b). There is a potential binding site of miR-33 in the 3′UTR of human *SCAP*. However, it is not conserved in mice and mRNA and protein levels of SCAP are the same in miR-33^+/+^ and miR-33^−/−^ mice (Supplementary Fig. S6a–c). Thus, the levels of precursor and mature forms of SREBP-1 are regulated in parallel.

### Reduction of SREBP-1 reverses fatty liver in miR-33-KO mice

**Figure 7:**
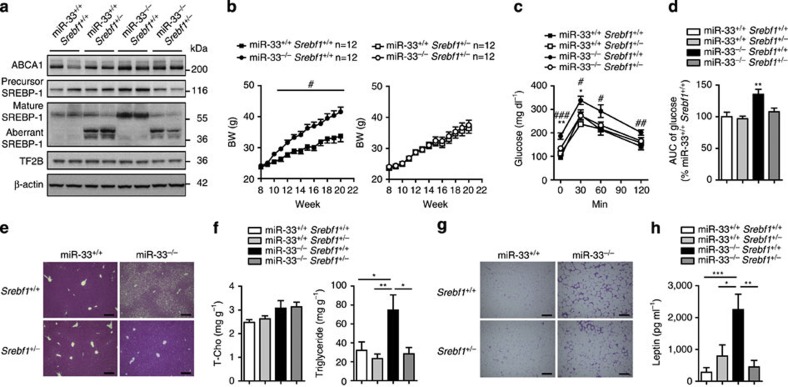
Reversal of hepatic steatosis by the reduction of SREBP-1 levels. (**a**) Western blotting analysis of SREBP-1 and ABCA1 levels in the livers of miR-33^+/+^*Srebf1*^+/+^, miR-33^+/+^*Srebf1*^+/−^, miR-33^−/−^*Srebf1*^+/+^ and miR-33^−/−^ *Srebf1*^+/−^ mice. Representative western blot images are shown (*n*=4). (**b**) Serial changes in BW levels of miR-33^+/+^*Srebf1*^+/+^, miR-33^+/+^*Srebf1*^+/−^, miR-33^−/−^*Srebf1*^+/+^ and miR-33^−/−^ *Srebf1*^+/−^ mice fed HFD under pair-feeding condition (*n*=12 each). (**c**) Serial changes in glucose levels after intraperitoneal injection of glucose into miR-33^+/+^*Srebf1*^+/+^, miR-33^+/+^*Srebf1*^+/−^, miR-33^−/−^*Srebf1*^+/+^ and miR-33^−/−^ *Srebf1*^+/−^ mice (*n*=11–12 each). *P<0.05 and **P<0.01 versus miR-33^−/−^*Srebf1*^+/−^ mice. ^#^*P*<0.05, ^##^*P*<0.01 and ^###^*P*<0.001 versus miR-33^+/+^*Srebf1*^+/+^ mice in one-way analysis of valiance test. (**d**) AUC of glucose levels in glucose tolerance tests in miR-33^+/+^*Srebf1*^+/+^, miR-33^+/+^*Srebf1*^+/−^, miR-33^−/−^*Srebf1*^+/+^ and miR-33^−/−^ *Srebf1*^+/−^ mice (*n*=11–12 each). **P<0.01 versus miR-33^−/−^*Srebf1*^+/−^ mice in one-way analysis of valiance test. (**e**) Representative microscopic images of the liver of miR-33^+/+^*Srebf1*^+/+^, miR-33^+/+^*Srebf1*^+/−^, miR-33^−/−^*Srebf1*^+/+^ and miR-33^−/−^ *Srebf1*^+/−^ mice fed HFD. Scale bars, 200 μm. (**f**) Cholesterol and triglyceride levels in the liver of miR-33^+/+^*Srebf1*^+/+^, miR-33^+/+^*Srebf1*^+/−^, miR-33^−/−^*Srebf1*^+/+^ and miR-33^−/−^ *Srebf1*^+/−^ mice fed HFD. *P<0.05 and **P<0.01 in one-way analysis of valiance test. (**g**) Representative microscopic images of the adipose tissue of miR-33^+/+^*Srebf1*^+/+^, miR-33^+/+^*Srebf1*^+/−^, miR-33^−/−^*Srebf1*^+/+^ and miR-33^−/−^ *Srebf1*^+/−^ mice fed HFD. Scale bars, 200 μm. (**h**) Serum leptin levels of miR-33^+/+^*Srebf1*^+/+^, miR-33^+/+^*Srebf1*^+/−^, miR-33^−/−^*Srebf1*^+/+^ and miR-33^−/−^ *Srebf1*^+/−^ mice fed HFD. *P<0.05, **P<0.01 and ***P<0.001 in one-way analysis of valiance test. Values are the means±s.e.m.

**Figure 8:**
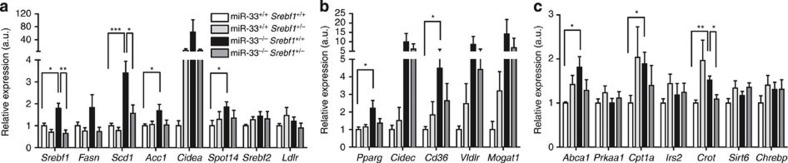
Relative mRNA expression levels of lipid metabolism-related genes. (**a**) *Srebf*s and lipogenic genes. *P<0.05, **P<0.01 and ***P<0.001 in one-way analysis of valiance test (*n*=6–8 each). (**b**) *Pparg* and its downstream genes. *P<0.05 in one-way analysis of valiance test (*n*=6–8 each). (**c**) Other lipid metabolism-related genes. *P<0.05 and **P<0.01 in one-way analysis of valiance test (*n*=6–8 each). Relative values of lipid metabolism-related genes in the livers of miR-33^+/+^*Srebf1*^+/+^, miR-33^+/+^*Srebf1*^+/−^, miR-33^−/−^*Srebf1*^+/+^ and miR-33^−/−^ *Srebf1*^+/−^ mice fed with HFD. Values are the means±s.e.m.

To elucidate the role of SREBP-1 in the phenotypic changes in miR-33^−/−^ mice fed HFD, we generated miR-33^−/−^ mice that have SREBP-1 expression levels similar to WT mice. As shown in Fig. 7a, protein levels of SREBP-1 are the same in miR-33^−/−^*Srebf1*^+/−^ and miR-33^+/+^*Srebf1*^+/+^ mice. Aberrant bands of SREBP-1 were observed in *Srebf1*-deficient mice (Fig. 7a and Supplementary Fig. S7a)^15^. mRNA levels of *Srebf1* in these mice are shown in Fig. 8a. Because miR-33^−/−^ mice showed positive palatability for HFD compared with miR-33^+/+^ mice (Fig. 4a), we analysed these four groups of mice under pair-feeding conditions. miR-33^+/+^*Srebf1*^+/−^, miR-33^−/−^*Srebf1*^+/+^ and miR-33^−/−^*Srebf1*^+/−^ mice received HFD in amounts that matched the rate of food intake of miR-33^+/+^*Srebf1*^+/+^ mice. As shown in Fig. 7b’s left panel, miR-33^−/−^*Srebf1*^+/+^ mice gained significantly more weight than miR-33^+/+^*Srebf1*^+/+^ mice under these conditions. Therefore, the BW gain in miR-33^−/−^ mice compared with miR-33^+/+^ mice is not caused by a change in food intake. The BW increase caused by miR-33 deficiency was abolished in a *Srebf1*^+/−^ background (Fig. 7b right and Supplementary Fig. S7b). On the other hand, glucose tolerance was ameliorated in miR-33^−/−^*Srebf1*^+/−^ compared with miR-33^−/−^*Srebf1*^+/+^ mice (Fig. 7c,d). There was no difference in BW or glucose tolerance among miR-33^+/+^*Srebf1*^+/+^, miR-33^+/+^*Srebf1*^+/−^ and miR-33^−/−^*Srebf1*^+/−^ mice (Fig. 7b–d). Serum insulin levels were reduced in miR-33^−/−^*Srebf1*^+/−^ mice compared with miR-33^−/−^*Srebf1*^+/+^ mice (Supplementary Fig. S7c, d). A striking difference was observed in the liver. Hepatic steatosis was reversed in miR-33^−/−^*Srebf1*^+/−^ mice compared with that in miR-33^−/−^*Srebf1*^+/+^ mice in both macro- and microscopic images and the liver triglyceride content of these mice was almost the same as that of miR-33^+/+^*Srebf1*^+/+^ and miR-33^+/+^*Srebf1*^+/−^ mice (Supplementary Fig. S7b and Fig. 7e,f). As shown in Fig. 7g, adipocyte size was partially reduced in miR-33^−/−^*Srebf1*^+/−^ mice compared with miR-33^−/−^*Srebf1*^+/+^ mice and there were still many infiltrating cells in miR-33^−/−^*Srebf1*^+/−^ mice. Serum leptin levels also reduced to baseline levels in miR-33^−/−^*Srebf1*^+/−^ mice (Fig. 7h). These results indicate that obesity and hepatic steatosis were ameliorated in miR-33^−/−^*Srebf1*^+/−^ mice compared with miR-33^−/−^*Srebf1*^+/+^ mice. There was no difference in the mRNA and protein levels of AMPKα and SIRT6, which are negative regulators of SREBP-1 and potential targets of miR-33, as shown in previous reports (Fig. 8c and Supplementary Fig. S7e). Finally, we examined the lipogenic gene profiles in the liver of these mice. As shown in Fig. 8a, the expression levels of *Srebf1* were significantly increased in miR-33^−/−^*Srebf1*^+/+^ mice compared with miR-33^+/+^*Srebf1*^+/+^ mice, and this was reversed in miR-33^−/−^*Srebf1*^+/−^ mice. The same pattern was observed in *Scd1*. Although they were not statistically significant, the expression levels of *Fasn*, *Acc1* and *Pparg* were reduced in miR-33^−/−^*Srebf1*^+/−^ mice compared with miR-33^−/−^*Srebf1*^+/+^ mice (Fig. 8a,b). Serum data for these mice are summarized in Supplementary Table S4. Serum ALP levels were significantly elevated in miR-33^−/−^*Srebf1*^+/+^ mice, which was reversed in miR-33^−/−^*Srebf1*^+/−^ mice.

## Discussion

In the current study, we showed that obesity and hepatic steatosis are observed in miR-33-deficient mice at the age of 50 weeks or when fed HFD for 12 weeks. We demonstrated that miR-33 targets SREBP-1, and miR-33^−/−^ mice had an enhanced expression of SREBP-1 in the liver. Study of miR-33^−/−^*Srebf1*^+/−^ mice clearly showed that enhanced expression of SREBP-1 caused obesity and fatty liver in miR-33^−/−^ mice (Supplementary Fig. S9a). These results indicate a previously unrecognized relationship between SREBP-1 and SREBP-2 through miR-33 (Supplementary Fig. S9b left).

Until now, there has been little evidence for an interaction between SREBP-1 and SREBP-2. It is known that in cholesterol-rich dietary conditions, SREBP-2 is downregulated at the cleavage level and SREBP-1c is transcriptionally activated through the activation of liver X receptors (LXRs) by the binding of oxysterols^16^,^17^,^18^,^19^. However, in sterol-depleted conditions, SREBP-2 is cleaved in the Golgi and the active N-terminal region translocates to the nucleus. Reduction in oxysterol levels leads to the inactivation of liver X receptors, which results in a decrease in SREBP-1c mRNA levels. Recently, it was shown that statin treatment induced hepatic miR-33 expression and at the same time decreased mRNA levels of miR-33 targets, including *Abca1* (ref. 8), *Abcb11* and *Atp8b1* (ref. 20) in mice. Thus, not only the activation by proteolytic cleavage but also its transcriptional regulation of SREBP-2/miR-33 is important *in vivo*. Our data showed that miR-33 targeted the 3′UTR of *Srebf1* and the upregulation of miR-33 by cholesterol depletion, considerably affecting the reduction of SREBP-1 expression. Therefore, based upon our findings that miR-33 regulates SREBP-1, miR-33 in the intron of *SREBP-2* may amplify reduction in SREBP-1 levels in sterol-depleted conditions (Supplementary Fig. S9b centre). SREBP-1c activates transcription of genes involved in fatty acid and triglyceride synthesis, such as genes encoding acetyl-CoA carboxylase, fatty acid synthase and elovl-6 and stearoyl-CoA desaturase. Therefore, it is possible that in sterol-depleted conditions, acetyl-CoA is preferred as a substrate for cholesterol production and not for fatty acid production, through the upregulation of miR-33. On the other hand, in cholesterol-rich conditions, miR-33 levels decrease^7^ and its negative regulation of SREBP-1 may be reduced. Thus, in this situation, acetyl-CoA is preferred as a substrate for fatty acid production (Supplementary Fig. S9b right).

Previously, several different SREBP-1 transgenic (TG) mice have been produced. Liver-specific transgenic mice using a phosphoenolpyruvate carboxykinase (PEPCK) promoter indicated that the livers of the SREBP-1a TG mice (PEPCK-SREBP-1a) were massively enlarged, owing to the accumulation of triglycerides and cholesterol^6^. PEPCK-SREBP-1c TG livers were only slightly enlarged with a moderate increase in triglycerides but not cholesterol. It is interesting that epididymal fat weight was not increased in these mice^6^. However, other liver-specific TG mice lines overexpressing human SREBP-1a and SREBP-1c under the control of the albumin promoter showed a vast accumulation of lipids in the liver and obesity as observed in miR-33^−/−^ mice^21^,^22^. Therefore, overexpression of SREBP-1 does not show consistent phenotypes and these changes may depend on the different promoters and expression patterns in organs of each transgenic line. Because SREBP-2 and miR-33 are expressed ubiquitously, miR-33^−/−^ mice may have mildly enhanced expression of SREBP-1 throughout the body. Previously developed SREBP-1 TG mice do not resemble miR-33-deficient mice in this context. Thus, obesity and hepatic steatosis developed slowly when fed NC and became prominent when fed HFD. Although SREBP-1c-deficient mice do not show any change in BW, it is possible that some compensatory mechanisms may have occurred in these mice^23^. Because miR-33^−/−^ mice showed a slight but significant increase in food intake when fed HFD, we conducted our crossbreeding experiments in pair-feeding conditions, which may have enabled us to clearly observe the changes. It is interesting that *Ldlr*^−/−^ mice that received anti-miR-33 oligonucleotides once a week for 14 weeks gained weight although the change was not statistically significant^24^,^25^. Therefore, reduction of miR-33 levels is related to unwanted consequences such as elevated BW. It should also be noted that the expression of *Pparg* and its downstream signalling molecules were enhanced in miR-33^−/−^*Srebf1*^+/+^ mice compared with WT control mice^26^,^27^,^28^,^29^. Because these molecules are attenuated in miR-33^−/−^*Srebf1*^+/−^ mice, enhanced expression of *Pparg* may be related to the increase in SREBP-1 levels associated with miR-33 deficiency^30^,^31^.

The phenotype of miR-33^−/−^*Srebf1*^+/−^ mice is not completely the same as that of WT mice, and this may be because of other miR-33 target genes. *Abca1* mRNA expression and serum HDL-C levels still tend to be higher in miR-33^−/−^*Srebf1*^+/−^ mice than those in miR-33^+/+^ *Srebf1*^+/+^ mice. Moreover, RIP140, another miR-33 target^12^,^32^, promotes the activity of NF-κB and upregulates the expression of genes implicated in inflammation such as TNFα and IL-6 in macrophages. Enhanced expression of RIP140 by miR-33 deficiency may affect the inflammatory conditions in adipose tissue.

It is important to explain why did not compare *Srebf1*^*+/+*^ with *Srebf1*^*+/−*^ mice, but only compared *miR-33*^*−/−*^*Srebf1*^*+/+*^ and *miR-33*^*−/−*^*Srebf1*^*+/−*^ in our experiments. The feedback system of SREBP-2 guarantees appropriate levels of cellular cholesterol. Meanwhile, excess glucose cumulatively activates SREBP-1 and increases triglyceride storage. The latter can be achieved by the fact that *Srebf1* expression is enhanced by SREBP-1 itself and that cleavage of SREBP-1 is less sensitive to sterol-suppression than SREBP-2 (refs 33, 34, 35, 36). Therefore, we hypothesize that the differences in the expression of SREBP-1 and its downstream molecules between *Srebf1*^+/+^ and *Srebf1*^*+/−*^ would be enhanced when SREBP-1 levels are increased by intercrossing with miR-33^*−/−*^ mice. The *Srebf1* levels are reduced in *Lep*^ob/ob^ × *Srebf1*^*+/−*^ mice compared with *Lep*^ob/ob^ mice^37^. However, in *Lep*^ob/ob^ mice, the *Srebf1* levels are not considerably enhanced in the liver, but are rather decreased in adipose tissue and the phenotype is different between *Lep*^ob/ob^ and miR-33^*−/−*^ mice. Because *Srebf1* may be more enhanced in miR-33^−/−^ mice than *Lep*^ob/ob^ mice, intercrossing with *Srebf1*^*+/−*^ had more effect in miR-33^−/−^ mice than *Lep*^ob/ob^ mice. Similarly, phenotypic difference between *Srebf1*^*+/+*^ and *Srebf1*^*+/−*^ mice with WT background may become small because *Srebf1* is not enhanced. Although a threshold may exist for the SREBP-1 levels that distinguishes between the normal condition and lipotoxicity caused by positive energy imbalance, the threshold level of SREBP-1 can only be achieved by feeding the *miR-33*^*−/−*^ *Srebf1*^*+/+*^ mice HFD or at an older age. This can also explain the fact that not much difference is observed between the *Srebf1*^*+/+*^ and *Srebf1*^*+/−*^ mice at the basal condition. For this reason, one copy of *Srebf1* can rescue the phenotype when miR-33 is absent. Recently, miRNAs have been recognized as therapeutic targets. It seems that it would be efficient to inhibit the function of a small number of miRNAs that have many different targets with similar functions at the same time. However, it is estimated that one miRNA may have hundreds of different target genes, and unpredicted results may be obtained by complete and long-term inhibition of an miRNA. It is also known that the results obtained by antisense oligonucleotide-based medicine are sometimes different from those obtained in miR-deficient mice. For example, the administration of an miR-21 antagomir prevented pressure-overload-induced cardiac hypertrophy and fibrosis in mice^38^; however, miR-21-deficient mice did not show any cardiac differences compared with WT mice under pressure overload^39^. As for miR-33, many target genes have been reported by computer algorithm and *in vitro* experiments such as luciferase-based 3′UTR analysis, only some of which show enhanced expression in miR-33-deficient mice.

A recent report indicated that the inhibition of miR-33a/b in non-human primates increases plasma HDL-C and lowers very-low-density lipoprotein triglycerides^40^. The authors showed a 50% decrease in *SREBF1* mRNA and protein in anti-miR-33-treated monkeys at 12 weeks. They speculated that the decrease in SREBP-1 may result from the derepression of negative regulators of this pathway such as AMPK, which is targeted by miR-33. They actually observed an increase in AMPK (*PRKAA1*) mRNA levels. However, there was no change in AMPKα levels in our experiment, and this point should be clarified in future experiments. It is true that in humans, and not in rodents, there is miR-33b in *SREBF1*. Because miR-33a and miR-33b have the same seed sequence, their targets would be the same. This may explain the differences in AMPKα levels. Further studies may be required to clarify whether there is autoregulation of SREBP-1 by miR-33b. In contrast, the experiment on monkeys was designed to administer antisense miR-33 for a limited time period. In the present study, we demonstrated that miR-33 deficiency serves to raise SREBP-1, increase fatty acid synthesis and promote fatty acid accumulation in the body. Therefore, long-term therapeutic modulation of miR-33 to cure metabolic diseases requires caution for obesity and related diseases, as miRNAs have potentially many target genes and we cannot detect all of them by computer analysis. Moreover, many genes are affected by these secondary or tertiary target genes. Careful observation of miR-deficient mice enables us to detect overall functions of miRNAs *in vivo*.

In conclusion, these results unravel a previously unrecognized interaction between SREBP-1 and SREBP-2 by the way of miR-33. It will be important to establish the tissue- and time-specific regulation of miR-33 to avoid unexpected side effects.

## Methods

### Materials

The antibodies used were anti-ABCA1 (NB400-105) (Novus Biologicals, Littleton, CO, USA), anti-GAPDH (14C10; no. 2118S), anti-IRS-2 (no. 4502S), anti-AMPKα (no. 2532), anti-SIRT6 (D8D12; no. 12486) (Cell Signaling Technology, Beverly, MA, USA), anti-β actin (AC-15; A5441, Sigma-Aldrich, St Louis, MO, USA), anti-SREBP-1 (sc-13551, sc-8984), anti-TF2B (sc-225), anti-PPARγ (sc-7273), anti-SCAP (sc-48671) (Santa Cruz Biotechnology, California, USA), antibodies. Anti-rabbit, anti-mouse and anti-goat IgG HRP-linked antibodies were purchased from GE Healthcare (Amersham, UK).*N*-acetyl-leu-leu-norleucinal (Calpain inhibitor; ALLN) and Complete Mini (Protease inhibitor cocktail) were from Roche. Pitavastatin (NK-104) was kindly provided by Kowa (Japan). *PPRE-*luciferase promoter plasmid (*PPRE*-luc) was kindly gifted by Dr Kelly-DP. *FAS-*luciferase promoter plasmid (*FAS-*luc) was obtained from Addgene. *SRE-*luciferase promoter plasmid (*SRE*-luc) was from Dr Yahagi-N. Mouse *Srebf1* was obtained from the FANTOM (functional annotation of the mouse) full-length mouse cDNA clone set, and Mouse *Srebf1* with or without the 3′UTR was cloned into pcDNA3.1.

### Cell culture

HepG2 cells were cultured Dulbecco’s modified Eagle’s medium (DMEM; Nacalai Tesque, Japan) supplemented with 10% fetal bovine serum (FBS). Mouse primary hepatocytes were obtained from male miR-33^+/+^ or miR-33^−/−^ mice at 8–10 weeks of age by the two-step collagenase perfusion method^41^. In brief, hepatocyte suspensions were obtained by passing collagenase type II (Gibco BRL, Life Technologies Inc., Rockville, MD, USA) digested liver through a 70 μm cell strainer, followed by centrifugation to collect the mature hepatocytes. After isolation, hepatocytes were resuspended in DMEM supplemented with 5% FBS, and seeded on collagen type I-coated dishes (Iwaki Asahi Glass Co. Ltd., Japan) at a density of 7 × 10^4^ cells ml^−1^. After incubation for 24 h, cells were used for experiments. For sterol-depleted experiments, cells were washed twice with DMEM without FBS two times and switched to DMEM containing 5% LPDS (Sigma-Aldrich) with or without pitavastatin (1 μM).

### Generation of miR-33^−/−^ *Srebf1* ^+/−^ mice

To obtain reduced levels of SREBP-1 in miR-33-deficient mice (miR-33^−/−^*Srebf1*^+/−^), miR-33^−/−^ mice were mated with *Srebf1*^+/−^ mice, which were a kind gift from Dr Shimano^15^. After being weaned at 4 weeks of age, mice were fed NC containing 4.5% fat until 8 weeks of age, and then switched to HFD (D12451; 45% fat by kcal; Research Diet Inc. New Brunswick, NJ, USA) or kept on NC for the next 12 weeks. All of the experimental protocols were approved by the Ethics Committee for Animal Experiments of Kyoto University. Primers for genotyping were as follows.

WT/KO (miR-33) sense; GGCACTACTTCTGATCCTTC

WT (miR-33) antisense; CAACTACAATGCACCACAGCTG

KO (miR-33) antisense; TTGGGATCCAGAATTCGTGATTAA

WT (*Srebf1*) sense; TGTGTCTGACCTGCAATCCT

WT (*Srebf1*) antisense; AGGCCAACACTAGTAGTCCATTG

KO (*Srebf1*) sense; AGGATCTCCTGTCATCTCACC

KO (*Srebf1*) antisense; GCCAACGCTATGTCCTGATA

### Western blotting

Western blotting was performed using standard procedures as described previously^42^. For cell experiments, ALLN (12.5 μg ml^−1^) was added to the cells 2 h before collection. Samples were lysed in lysis buffer consisting of 100 mM Tris-HCl, pH 7.4, 75 mM NaCl and 1% Triton X-100 (Nacalai Tesque). The lysis buffer was supplemented with complete mini protease inhibitor (Roche), ALLN (25 μg ml^−1^), 0.5 mM NaF and 10 μM Na_3_VO_4_ just before use. Nuclear protein was extracted using the CelLytic NuCLEAR Extraction Kit (Sigma-Aldrich) in accordance with the manufacturer’s instructions. The protein concentration was determined using a bicinchoninic acid (BCA) protein assay kit (Bio-Rad). All samples (20 μg of protein) were suspended in lysis buffer, fractionated using NuPAGE 4–12% Bis-Tris (Invitrogen) gels and transferred to a Protran nitrocellulose transfer membrane (Whatman). The membrane was blocked using 1 × phosphate-buffered saline (PBS) containing 5% non-fat milk for 1 h and incubated with the primary antibody (anti-ABCA1; 1:1,000, anti-ABCG1; 1:1,000, anti-IRS-2; 1:500, anti-AMPKα; 1:1,000, anti-SREBP-1; 1:250 (Supplementary Fig. S10), anti-TF2B; 1:1,000, anti-PPARγ; 1:250, anti-β actin; 1:3,000, anti-GAPDH; 1:3,000, anti-SIRT6; 1:1,000 and anti-SCAP; 1:200) overnight at 4 °C. Following a washing step in PBS-0.05% Tween 20 (0.05% T-PBS), the membrane was incubated with the secondary antibody (anti-rabbit, anti-mouse or anti-goat IgG HRP-linked; 1:2,000) for 1 h at 4 °C. The membrane was then washed in 0.05% T-PBS and detected by ECL Western Blotting Detection Reagent (GE Healthcare), using an LAS-1000 system (Fuji Film). Full-length images on immunoblots are shown in Supplementary Figs S11–S13.

### RNA extraction and qRT–PCR

Total RNA was isolated and purified using TriPure Isolation Reagent (Roche), and cDNA was synthesized from 1 μg of total RNA using the Transcriptor First Strand cDNA Synthesis Kit (Roche) in accordance with the manufacturer’s instructions. For quantitative RT–PCR, specific genes were amplified by 40 cycles using SYBR^**™**^ Green PCR Master Mix (Applied Biosystems). Expression was normalized to the housekeeping gene β-actin. Gene-specific primers are listed in Supplementary Table S1.

### Quantitative PCR for miRNAs

Total RNA was isolated using TriPure Isolation Reagent (Roche). miR-33 was measured in accordance with the TaqMan MicroRNA assays (Applied Biosystems) protocol, and the products were analysed using a thermal cycler (ABI Prism7900HT sequence detection system). Samples were normalized by U6 snRNA expression.

### Measurement of fatty acid synthesis

Following 4 h of fasting, mice were intraperitonially injected with 10 μCi [1-^14^C]-sodium acetate (PerkinElmer Co., Ltd.). Male mice at 10 weeks of age were sacrificed 30 min after injection and livers were rinsed in ice-cold 1 × PBS. Liver samples were saponified by heating in 3 ml of 30% KOH (w/v) at 70 °C for 15 min, followed by the addition of 3 ml of 95% ethanol (v/v) and continued heating at 70 °C for a further 2 h. Saponified fatty acids were acidified with 3 ml of 9 M H_2_SO_4_ and extracted with petroleum ether^13^,^14^.

### Dual luciferase assays

Full-length PCR fragments of the 3′UTR of *SREBF1* were amplified from human or mouse cDNAs and subcloned downstream of a CMV-driven Firefly luciferase cassette in a pMIR-REPORT vector (Ambion). To create WT or mutant 3′UTR luciferase reporter genes, a fragment of the *Srebf1* 3′UTR as follows was inserted into a pMIR-REPORT vector:

Wild type;

CTTCCAAAACAATCGTGGTATCTTTATTGACTTTTTTTTTTCTGAATGCAATGACTGTTTTTTTTTTTTTTAAC

Mutant;

CTTCCAAAACAATCGTGGTATCTTTATTGACTTTTTTTTTTCTGA***TACGT***ATGACTGTTTTTTTTTTTTTTAAC

For *SRE* and *FAS* promoter assay, 293T cells were co-transfected with mouse *Srebf1* with full-length 3′UTR or without 3′UTR, along with expression plasmids for miR-control (negative control), or miR-33. An internal control reporter, *Renilla reniformis* luciferase, driven by the thymidine kinase (TK) promoter (pRL-TK: Promega) was also co-transfected to normalize the transfection efficiency. Luciferase activities were measured using a dual luciferase kit (PicaGene dual kit, Toyo Ink Co.). The relative luciferase activity of each construct (arbitrary unit) was reported as the fold induction.^42^

### Lentivirus production and DNA transduction

We produced lentiviral stocks in 293FT cells in accordance with the manufacturer’s protocol (Invitrogen). In brief, virus-containing medium was collected 48 h post transfection and filtered through a 0.45-μm filter. One round of lentiviral infection was performed by replacing the medium with virus-containing medium (containing 8 μg of Polybrene per ml), followed by centrifugation at 2,500 rpm for 30 min at 32 °C. Cells were used for analysis 3 days after DNA transduction.

### IPGTT and insulin tolerance test

For IPGTT, after overnight fasting, male mice at 20 or 50 weeks of age were injected with 1.5 g kg^−1^ glucose intraperitoneally. For insulin tolerance test, after a 4-h fast, mice were injected intraperitoneally with insulin (0.75 u kg^−1^ and 1.0 u kg^−1^ for NC and HFD, respectively, Humulin R; Eli Lilly Japan KK). Blood was obtained from the orbital vein and glucose levels were measured using a glucose sensor.

### Measurement of serum insulin and leptin levels

We quantified serum levels of insulin and leptin in male mice at 20 weeks of age using an ELISA assay kit for mouse insulin and leptin in accordance with the manufacturer’s instructions (Shigayagi Co. Ltd, Shibukawa, Japan).

### Biochemical analysis of serum

After mice (male at 20 or 50 weeks of age) were fasted for 4–6 h, blood was obtained from the inferior vena cava of anaesthetised mice, and serum was separated by centrifugation at 4 °C and stored at −80 °C. Biochemical data were measured by standard methods using a Hitachi 7180 Auto Analyzer (Nagahama Life Science Laboratory, Nagahama, Japan).

### Measurement of cholesterol and triglyceride in the liver

Lipids in the liver were extracted by the Folch procedure^43^. In brief, lipids were extracted by addition of ice-cold MeOH followed by the addition of ice-cold CHCl_3_. High purity water was added and the sample kept on ice for an additional 10 min with occasional mixing. The sample was centrifuged for 5 min at 2,000 *g* and the upper (aqueous) phase was removed and reextracted by addition of ice-cold CHCl_3_:MeOH (2:1, v/v) as above. The upper phase was discarded and both organic phases were combined, dried under nitrogen stream. Lipids were quantified using standard enzymatic colorimetric methods (Sky Light Biotech, Akita, Japan).

### Measurement of fat body mass by CT

CT scans were obtained and fat body mass and lean body mass were analysed using a Lathea LTC-100 (Aloka, Tokyo, Japan) under pentobarbital anaesthesia.

### DNA microarray analysis

Five liver RNA samples from miR-33^+/+^ or miR-33^−/−^ male mice at 16 weeks of age receiving NC were pooled and analysed using a DNA microarray (3D-Gene Mouse Oligo chip 24 k, Toray, Tokyo, Japan).

### Pair feeding

Every other day, male mice received the same amount of food consumed by miR-33^+/+^*Srebf1*^+/+^ mice (WT) on the previous 2 days, from 8 to 20 weeks of age.

### Assessment of metabolic rate and activity

Oxygen consumption and activity of male mice at 16 weeks of age were measured with an indirect calorimetric system. In brief, room air was pumped through an acrylic metabolic chamber, and the expired gas was filtered through thin cotton, dried and subjected to gas analysis (model RL-600; Alco System, Tokyo, Japan)^44^.

### Statistics

Data are presented as means±s.e.m. Statistical comparisons were performed using unpaired two-tailed Student’s *t*-tests or a one-way analysis of variance with the Bonferonni *post hoc* test where appropriate, with a probability value of <0.05 taken to indicate significance.

## Author contributions

T.H., T.N. and K.O. designed the project; T.H., T.N., O.B., Y.K., T.N., M.N., S.U., M.I., M.S. and S.M. performed experiments; T.H., T.N., N.Y., H.S., K.I., H.M., T.M., K.H., N.K., M.Y., T.K., T.K. and K.O. analysed and interpreted data; N.Y. and H.S. contributed materials; and T.H., T.N. and K.O. prepared the manuscript.

## Additional information

**How to cite this article:** Horie, T. *et al.* MicroRNA-33 regulates sterol regulatory element-binding protein 1 expression in mice. *Nat. Commun.* 4:2883 doi: 10.1038/ncomms3883 (2013).

## Supplementary Material

Supplementary Information Supplementary Figures S1-S13 and Supplementary Tables S1-S4

## Figures and Tables

**Table 1:** Serum profile of miR-33^+/+^ and miR-33^*−/−*^ on NC or HFD.

	**miR-33**^**+/+**^ **NC (*****n*****=8)**	**miR-33**^**−/−**^ **NC (*****n*****=8)**	**miR-33**^**+/+**^ **HFD (*****n*****=5)**	**miR-33**^*−/−*^ **HFD (*****n*****=5)**
AST (IU l^−1^)	67.63±5.43	56.50±11.89	53.80±2.89	134.40±18.48**
ALT (IU l^−1^)	39.38±6.02	41.00±11.95	31.40±3.92	167.40±29.15**
ALP (IU l^−1^)	186.88±15.29	238.13±14.57*	138.80±9.83	210.60±20.79*
T-CHO (mg dl^−1^)	85.75±3.83	110.25±6.16**	157.40±13.25	244.8±21.42**
TG (mg dl^−1^)	35.63±4.39	34.50±4.82	21.80±2.33	17.60±3.72
NEFA (μEq l^−1^)	766.8±30.89	867.1±53.68	778±61.21	979±55.40*
LDL-C (mg dl^−1^)	5.50±0.63	6.88±0.72	11.40±0.51	26.00±5.30*
HDL-C (mg dl^−1^)	52.38±2.08	66.13±2.72**	79.00±4.71	83.60±1.25

Values are the means±s.e.m. Statistical comparisons were made by Student’s *t*-test (*P<0.05, **P<0.01).
