# Supplementary material for: MicroRNA-33 regulates sterol regulatory element-binding protein 1 expression in mice
Source: Nat Commun. 2013 Dec 3;4:2883. doi: 10.1038/ncomms3883 (PMC3863899; doi:10.1038/ncomms3883)
Supplement: Supplementary Information — Supplementary Figures S1-S13 and Supplementary Tables S1-S4 [file ncomms3883-s1.pdf]

# Supplementary Information

## MicroRNA-33 regulates sterol regulatory element-binding protein 1 expression in mice

**Takahiro Horie, Tomohiro Nishino, Osamu Baba, Yasuhide Kuwabara, Tetsushi Nakao, Masataka Nishiga, Shunsuke Usami, Masayasu Izuhara, Naoya Sowa, 1Naoya Yahagi, Hitoshi Shimano, Shigenobu Matsumura, Kazuo Inoue, Hiroyuki Marusawa, Tomoyuki Nakamura, Koji Hasegawa, Noriaki Kume, Masayuki Yokode, Toru Kita, Takeshi Kimura, and Koh Ono**

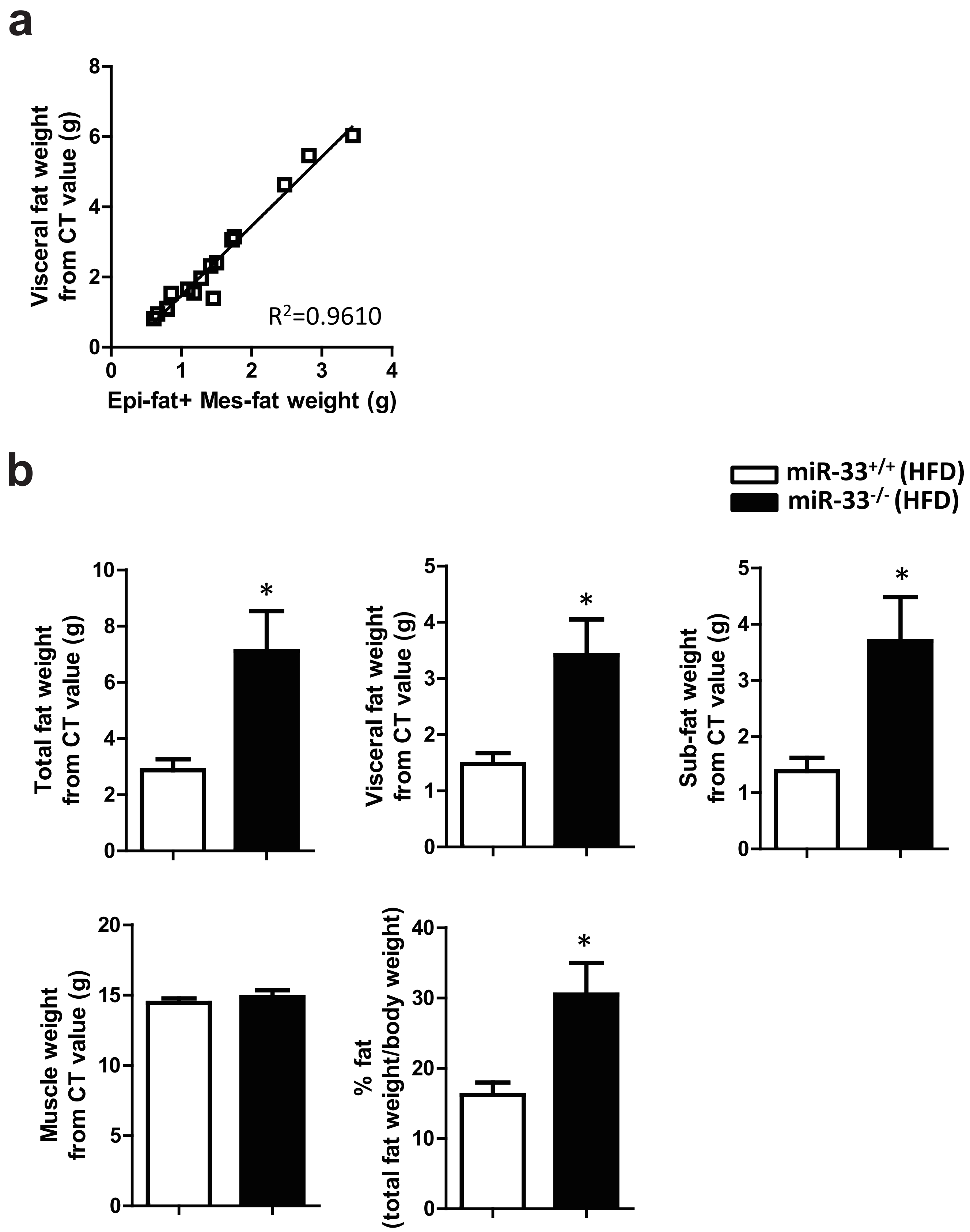


**Supplementary Figure S1. Estimation of fat weight from CT images.**

**(a)** Relationship between estimated fat weight and actual weight. **(b)** Estimated fat weight of miR-33$^{+/+}$ and miR-33$^{-/-}$ mice fed HFD. Values are the means ± s.e.m. (n = 8 each, *p<0.05 in Student's t-test).

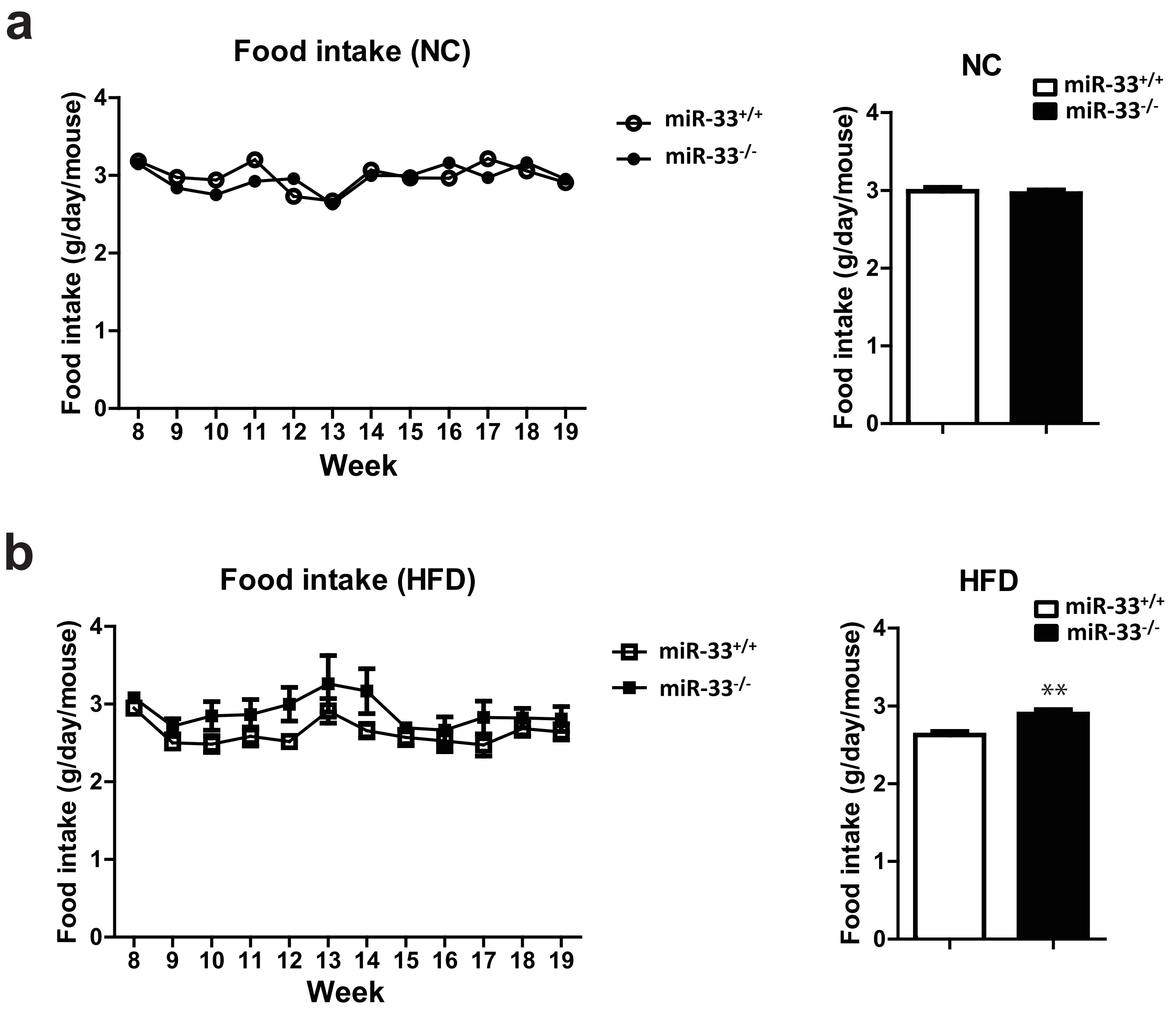


**Supplementary Figure S2. Food intake of miR-33$^{+/+}$ and miR-33$^{-/-}$ mice.**

**(a)** Serial changes in food intake associated with NC feeding. **(b)** Serial changes of food intake associated with HFD feeding. Values are the means ± s.e.m. Statistical comparisons were made by Student's t-test (n=5, **p < 0.01).

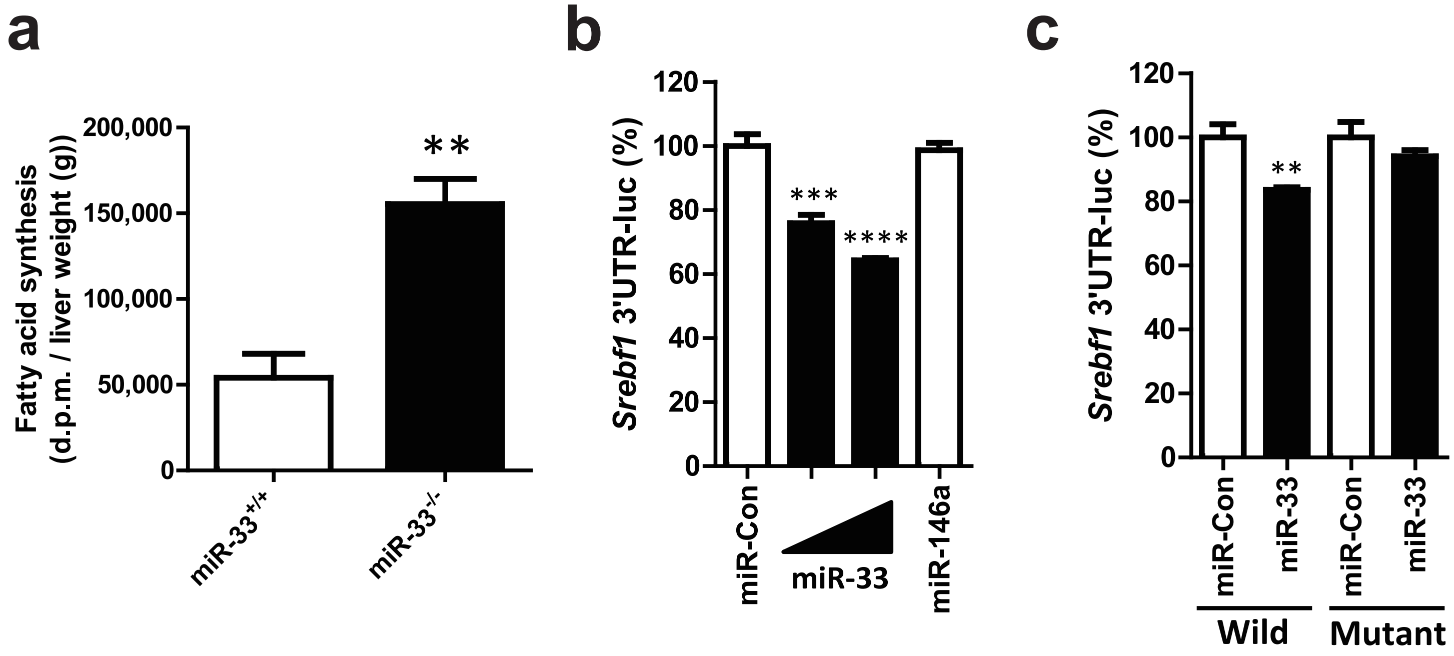


**Supplementary Figure S3. miR-33 deficiency increased fatty acid synthesis *in vivo* and miR-33 targets the 3’UTR of *Srebf1* in COS-7 cells.**

**(a)** *De novo* fatty acid synthesis in the livers of miR-$33^{+/+}$ and miR-$33^{-/-}$ mice (n = 4 each, **p < 0.01 in Student’s t-test). **(b)** Luciferase reporter activity of mouse *Srebf1* 3′UTR in COS-7 cells. miR-control (miR-Con) and miR-146a were used as negative controls (n = 4 each, ***p < 0.001, ****p < 0.0001 in one-way analysis of valiance test) **(c)** Luciferase reporter activity of wild-type or mutant *Srebf1* 3′UTR at the potential miR-33 binding site in COS-7 cells (n = 4 each, **p < 0.01 in Student’s t-test). Values are the mean ± s.e.m.

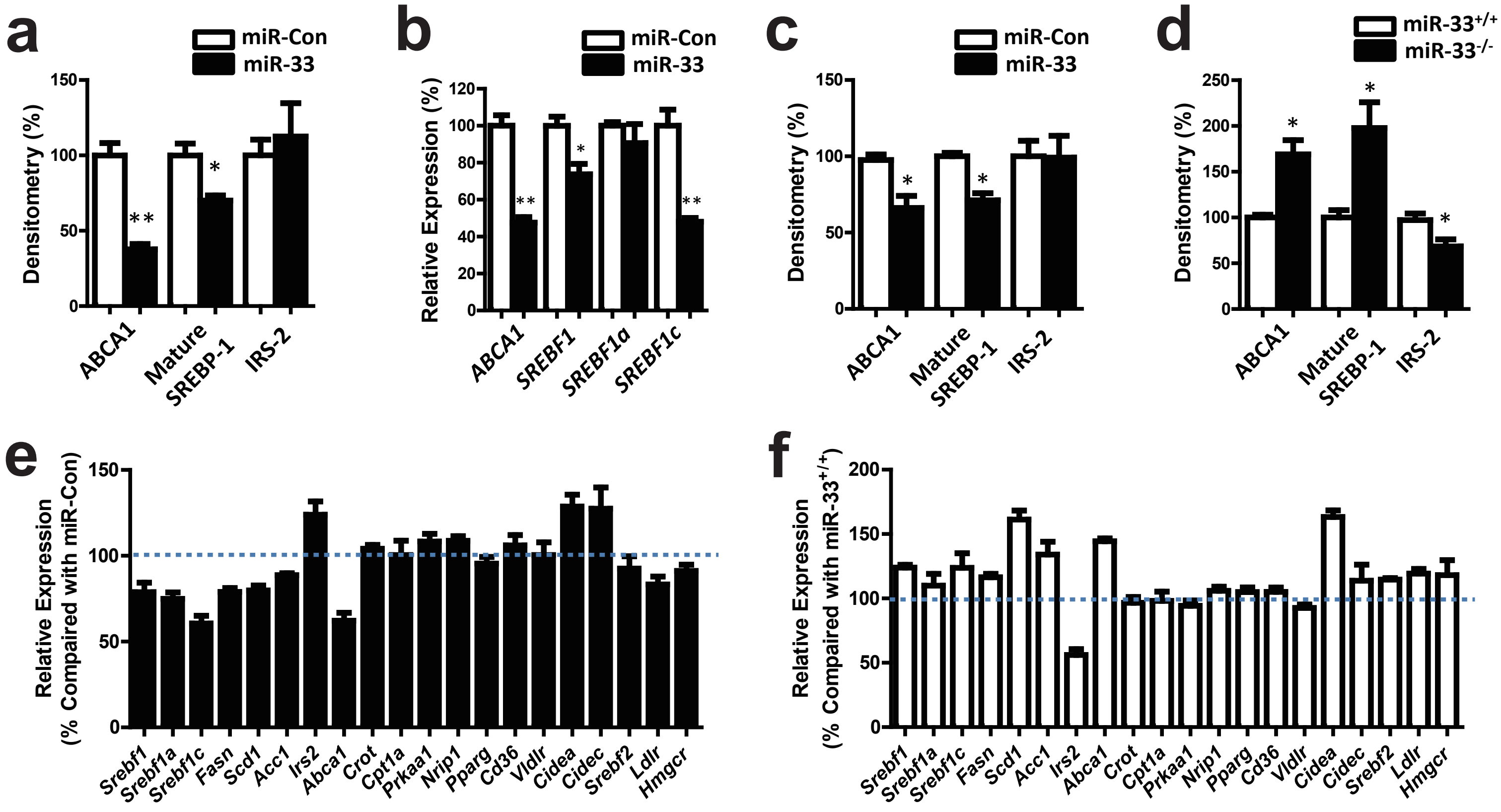


**Supplementary Figure S4. The expressions of lipid metabolism-associated genes in HepG2 cells and primary hepatocytes transduced with miR-Con or miR-33 expression vector and in miR-33$^{+/+}$ and miR-33$^{-/-}$ mice.**

**(a)** ABCA1, SREBP-1, and IRS-2 expression levels in HepG2 cells transduced with miR-Con or miR-33 expression vector. **(b)** *ABCA1*, *SREBF1*, *SREBF1a*, and *SREBF1c* expression levels in HepG2 cells transduced with miR-Con or miR-33 expression vector *p<0.05, **p<0.01. **(c)** ABCA1, SREBP-1, and IRS-2 expression levels in primary hepatocytes transduced with miR-Con or miR-33 expression vector. *p<0.05. **(d)** ABCA1, SREBP-1, and IRS-2 expression levels in primary hepatocytes prepared from miR-33$^{+/+}$ and miR-33$^{-/-}$ mice. *p<0.05. **(e)** Relative expression levels of lipid metabolism-related genes in primary hepatocytes transduced with miR-Con or miR-33 expression vector. **(f)** Relative expression levels of lipid metabolism-related genes in primary hepatocytes prepared from miR-33$^{+/+}$ and miR-33$^{-/-}$ mice. Values are the means ± s.e.m. Statistical comparisons were made by Student's t-test.

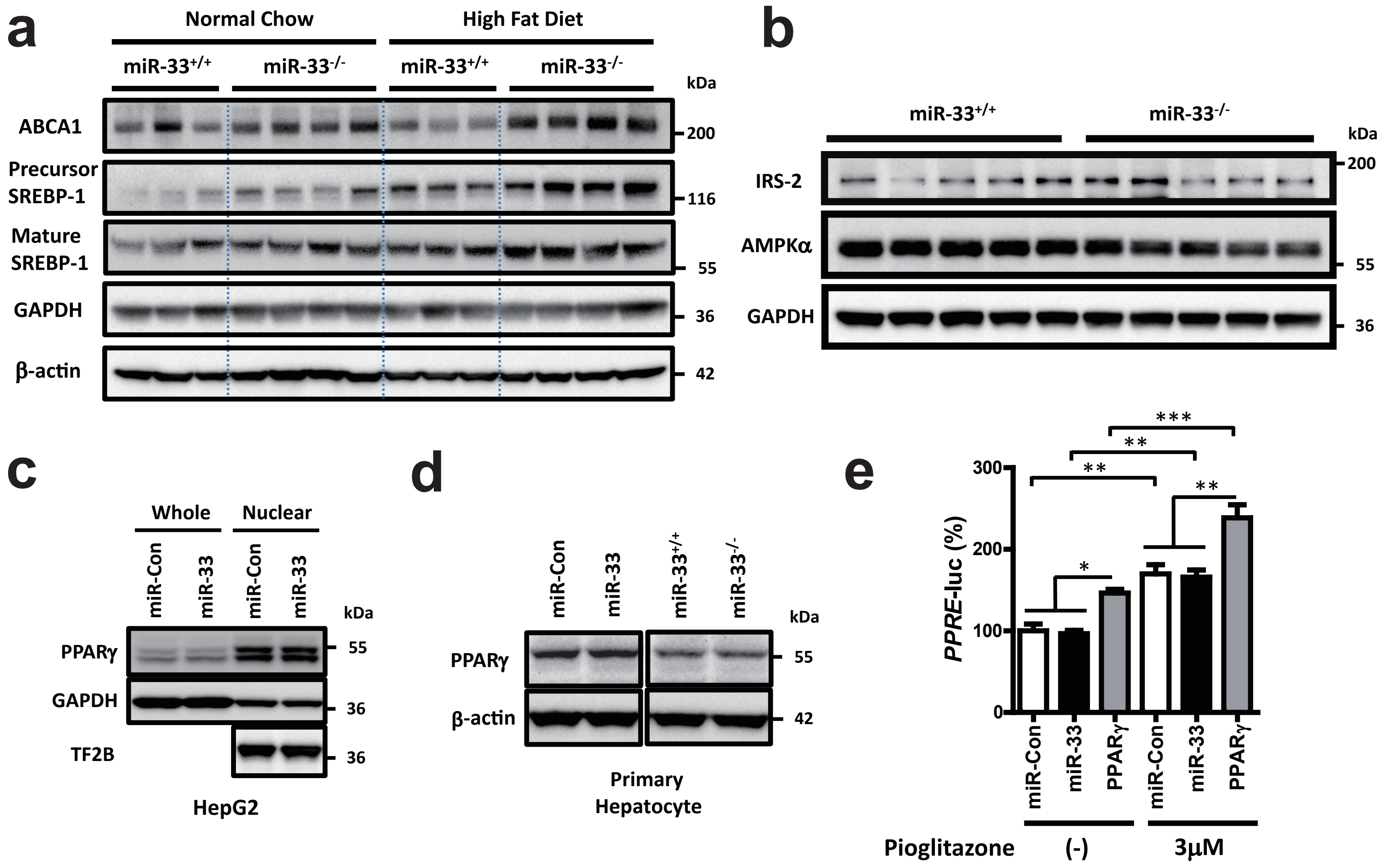


**Supplementary Figure S5. Western blotting analysis of ABCA1, SREBP-1, IRS-2, AMPKα, and PPARγ.**

**(a)** ABCA1 and SREBP-1 protein levels in the livers of miR-$33^{+/+}$ and miR-$33^{-/-}$ mice fed NC or HFD. **(b)** AMPKα and IRS-2 protein levels in the livers of miR-$33^{+/+}$ and miR-$33^{-/-}$ mice. **(c)** PPARγ protein levels in HepG2 cells transduced with miR-Con or miR-33 expression vector. **(d)** PPARγ protein levels in primary hepatocytes transduced with miR-Con or miR-33 expression vector and primary hepatocytes prepared from miR-$33^{+/+}$ and miR-$33^{-/-}$ mice. **(e)** Luciferase reporter activity of *PPRE* in HepG2 cells. HepG2 cells with or without 3 μM pioglitazone treatment were transfected with the *PPRE* luciferase construct, along with expression plasmids for miR-Con (negative control), miR-33, or PPARγ (positive control). Values are the mean ± s.e.m. Statistical comparisons were made by one-way analysis of valiance test (n = 4 each, $^{*}p < 0.05$, $^{**}p < 0.01$, $^{***}p < 0.001$).

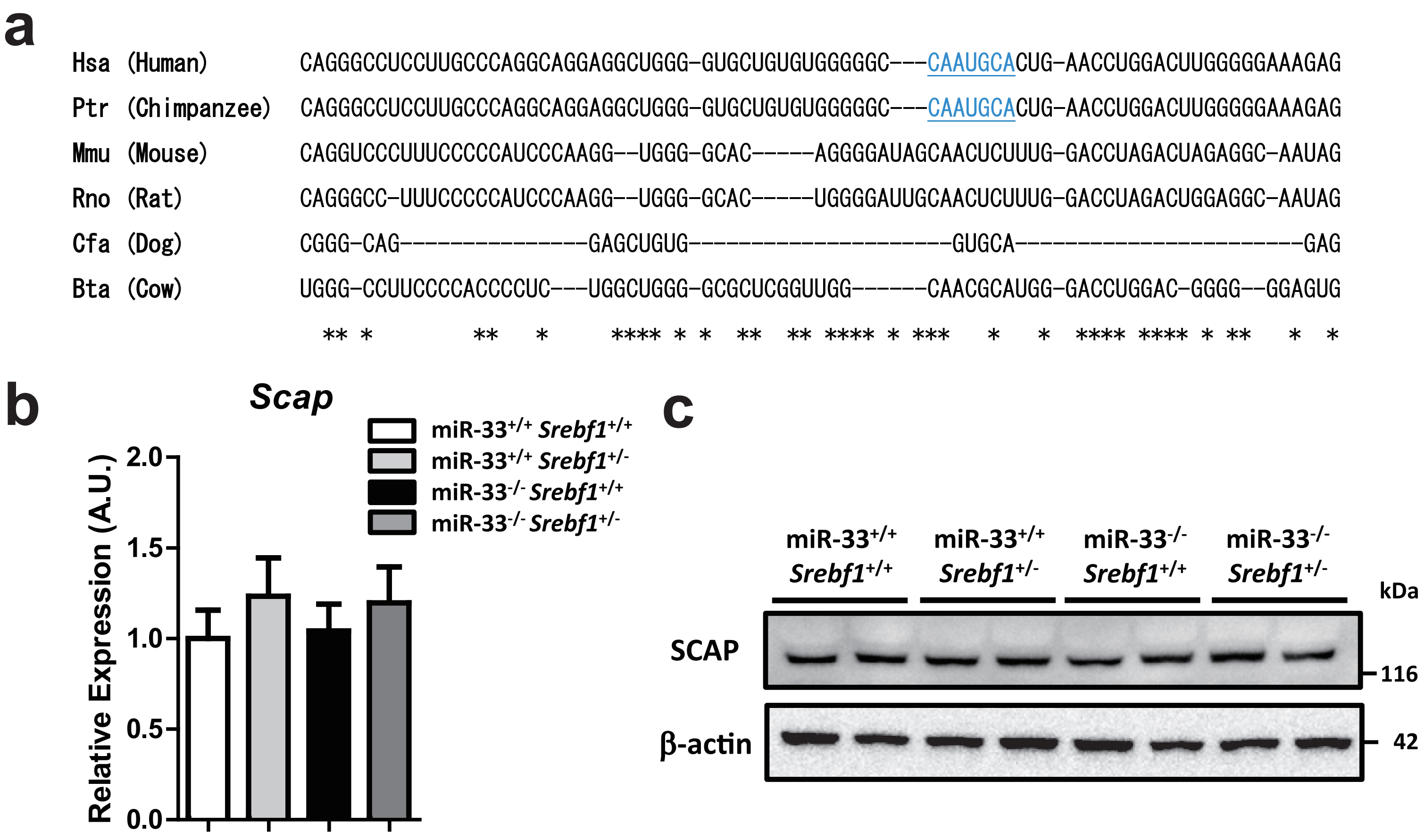


**Supplementary Figure S6. The expression of SCAP in miR-33$^{+/+}$*Srebf1*$^{+/+}$, miR-33$^{+/+}$*Srebf1*$^{+/−}$, miR-33$^{−/−}$*Srebf1*$^{+/+}$, and miR-33$^{−/−}$ *Srebf1*$^{+/−}$ mice fed HFD.**

**(a)** Sequence alignment of *Scap* 3′UTR at the potential miR-33 binding site. * indicates the conservation among species. **(b)** Quantitative real-time PCR analysis of *Scap*. Values are the mean ± s.e.m. (n = 6−8 each). **(c)** Western blotting analysis of SCAP levels. β-actin was used as a loading control.

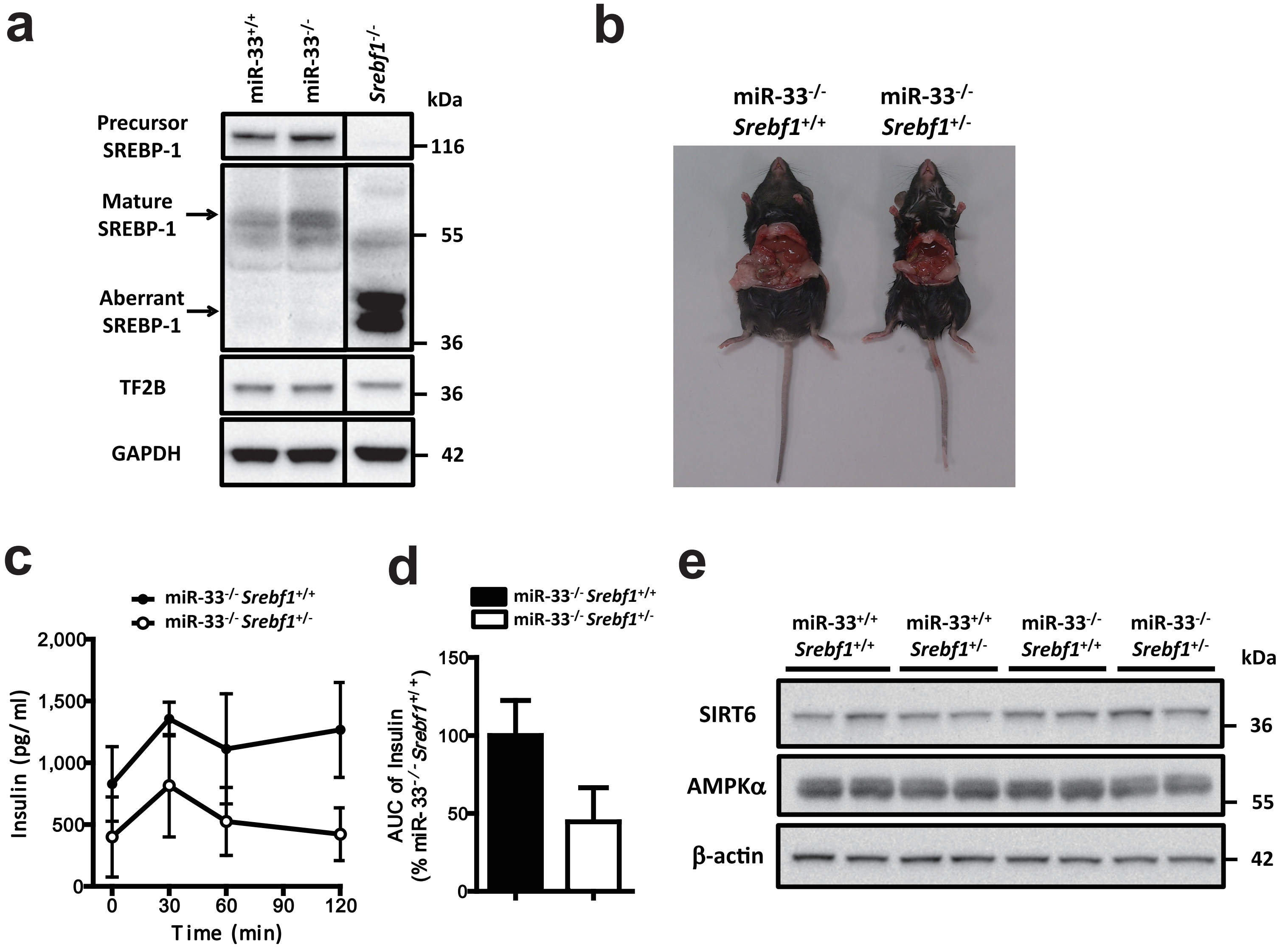


**Supplementary Figure S7. Phenotypic changes by the reduction of SREBP-1 levels in miR-33$^{-/-}$ mice.**

**(a)** Western blotting analysis of SREBP-1 in the liver of miR-33$^{+/+}$, miR-33$^{-/-}$ and *Srebf1*$^{-/-}$ mice fed NC. **(b)** Representative picture of miR-33$^{-/-}$*Srebf1*$^{+/+}$ and miR-33$^{-/-}$*Srebf1*$^{+/-}$ mice fed HFD. **(c)** Serial changes of insulin levels after intraperitoneal injection of glucose in miR-33$^{-/-}$*Srebf1*$^{+/+}$ and miR-33$^{-/-}$*Srebf1*$^{+/-}$ mice fed HFD (n = 5 each). **(d)** AUC of insulin levels after intraperitoneal injection of glucose in miR-33$^{-/-}$*Srebf1*$^{+/+}$ and miR-33$^{-/-}$*Srebf1*$^{+/-}$ mice fed HFD (n = 5 each). **(e)** Western blotting analysis of SIRT6 and AMPKα in miR-33$^{+/+}$*Srebf1*$^{+/+}$, miR-33$^{+/+}$*Srebf1*$^{+/-}$, miR-33$^{-/-}$*Srebf1*$^{+/+}$, and miR-33$^{-/-}$*Srebf1*$^{+/-}$ mice fed HFD. β-actin was used as a loading control. Values are the means ± s.e.m.

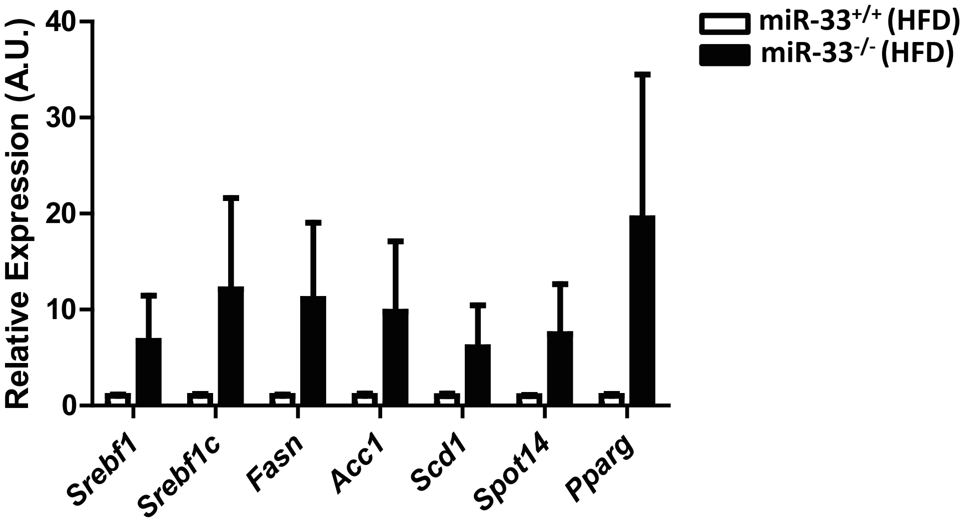


**Supplementary Figure S8. Relative mRNA expression levels of lipid metabolism -related genes in epididymal fat pads from miR-33$^{+/+}$ and miR-33$^{−/−}$ mice fed HFD.** Values are the means ± s.e.m. (n=5).

a

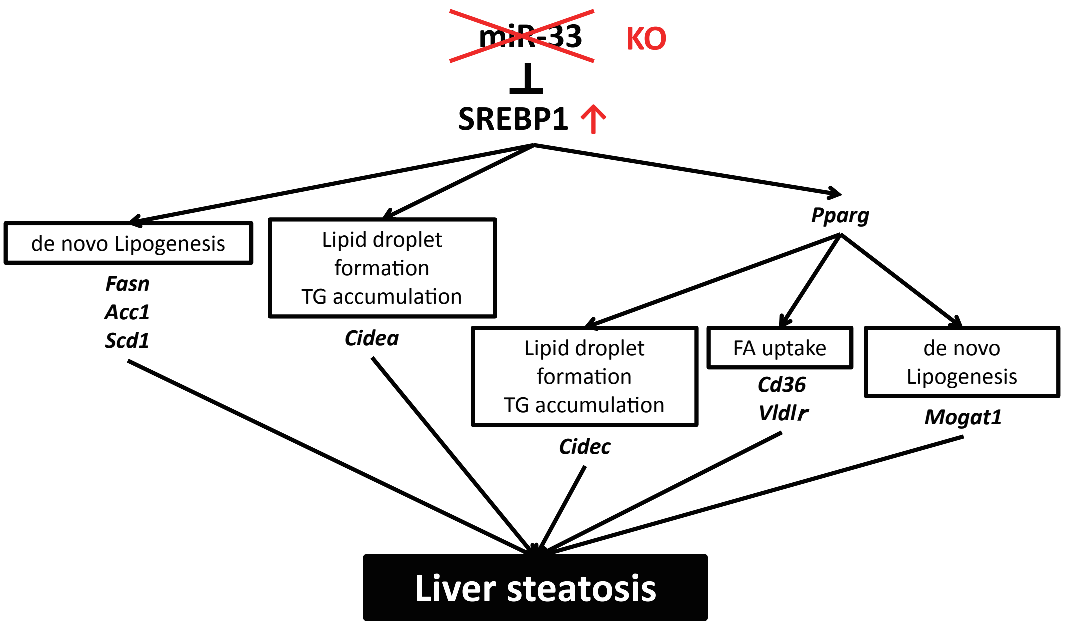


b

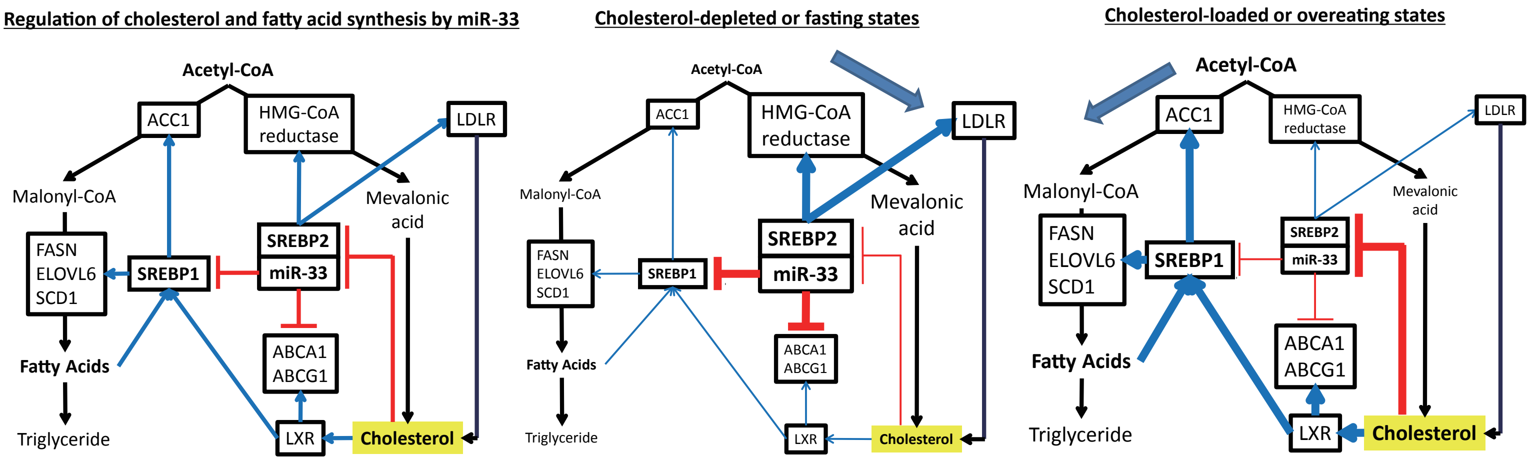


**Supplementary Figure S9. Schematic overview of the function of miR-33 in the regulation of SREBP-1 expression levels.**

**(a)** Overview of the function of miR-33 deficiency on liver steatosis.

**(b)** Context-dependent change of the role of miR-33 on lipid metabolism.

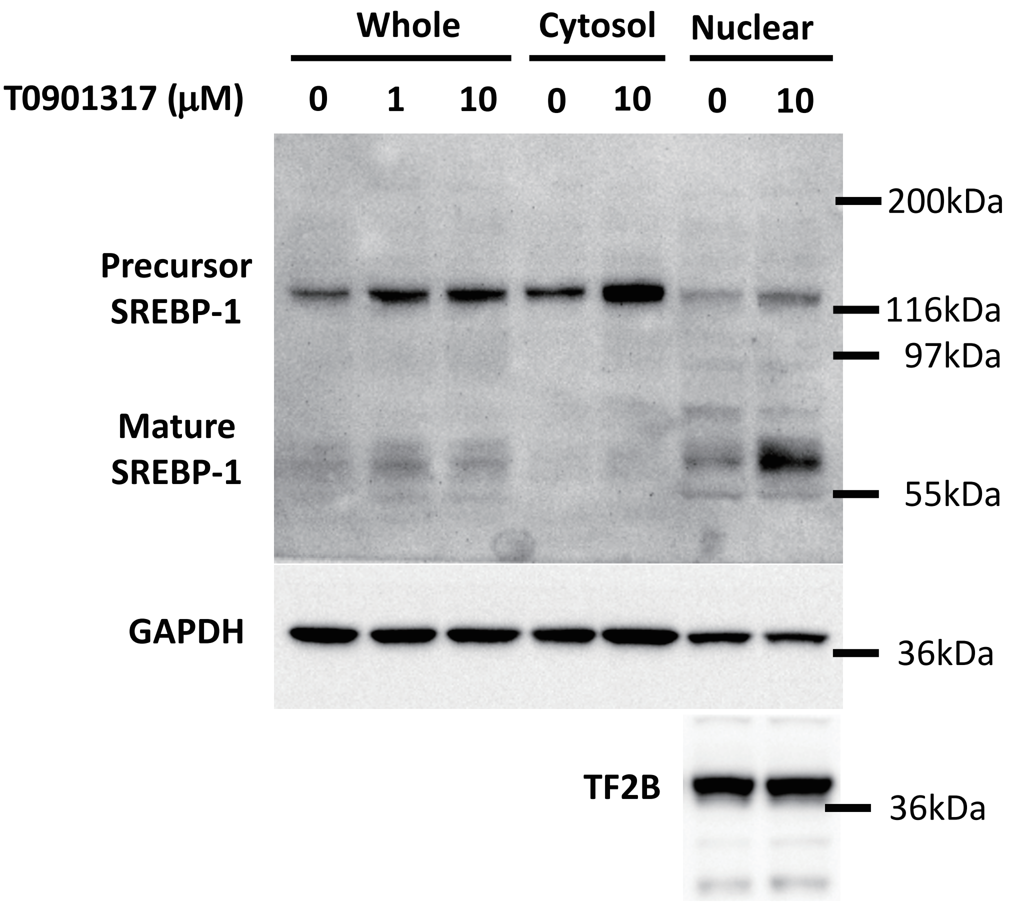


**Supplementary Figure S10. Western blotting for SREBP-1, GAPDH, TF2B in T0901317-stimulated HepG2 cells.**

HepG2 cells were stimulated with the indicated concentrations of T0901317 for 24 h.

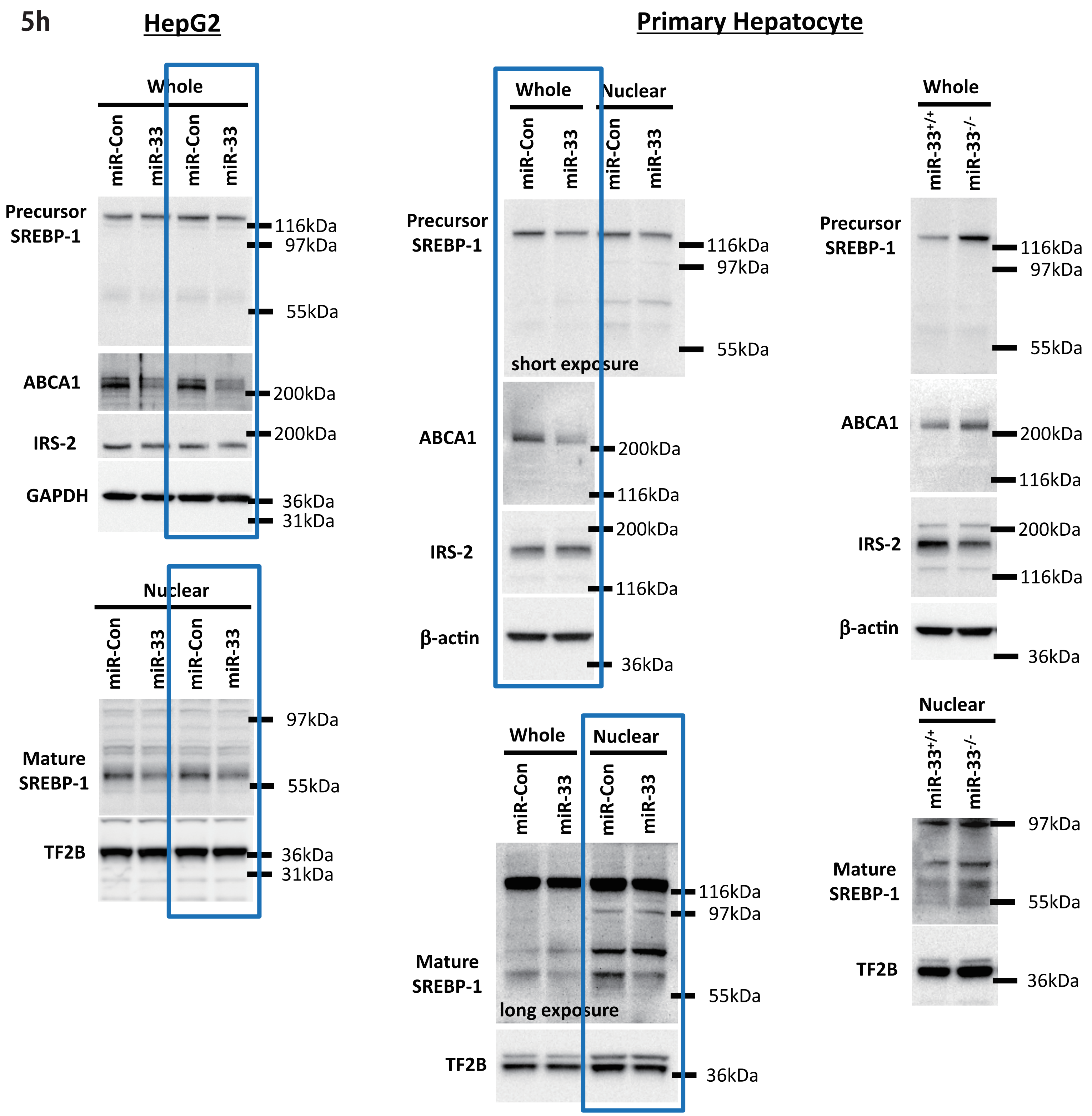


**Supplementary Figure S11. Scans of the original blots in Figure 5h**

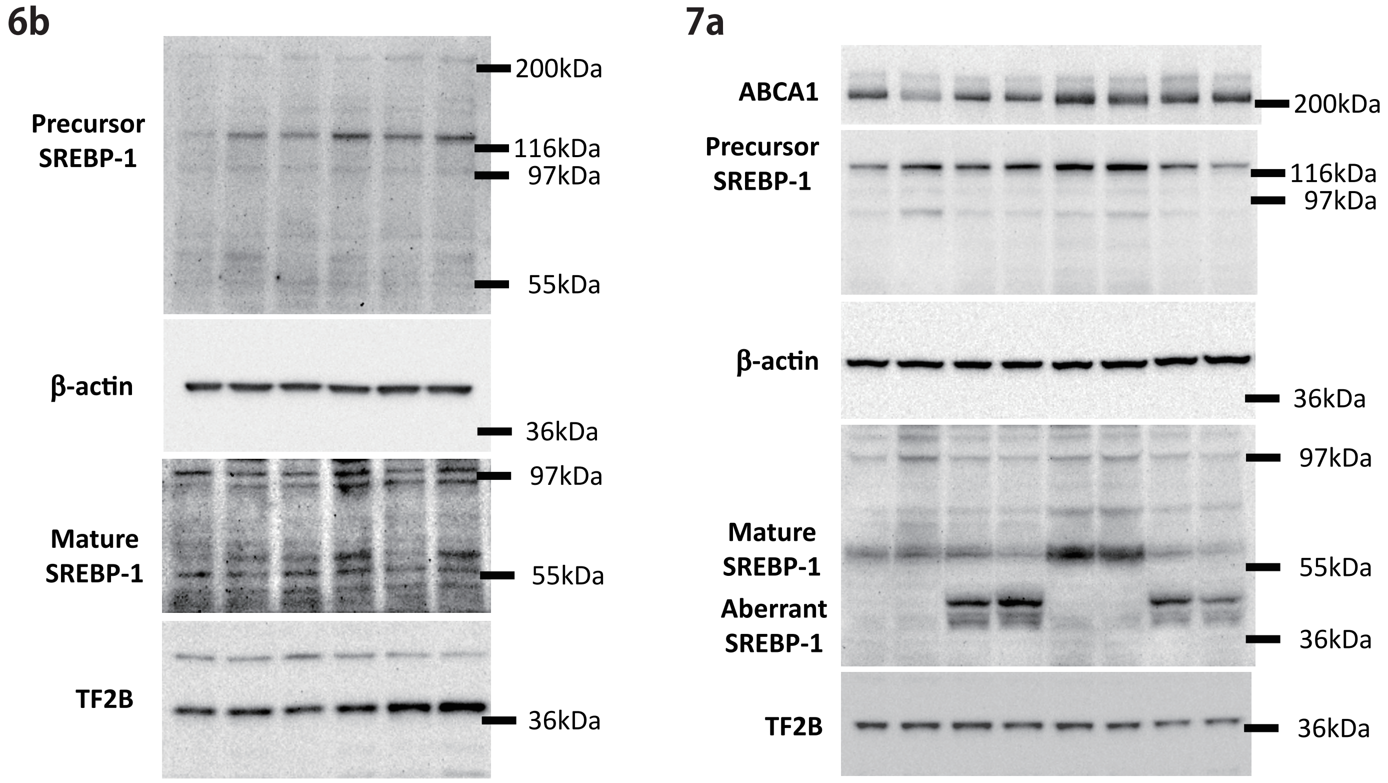


**Supplementary Figure S12. Scans of the original blots in Figure 6b and 7a**

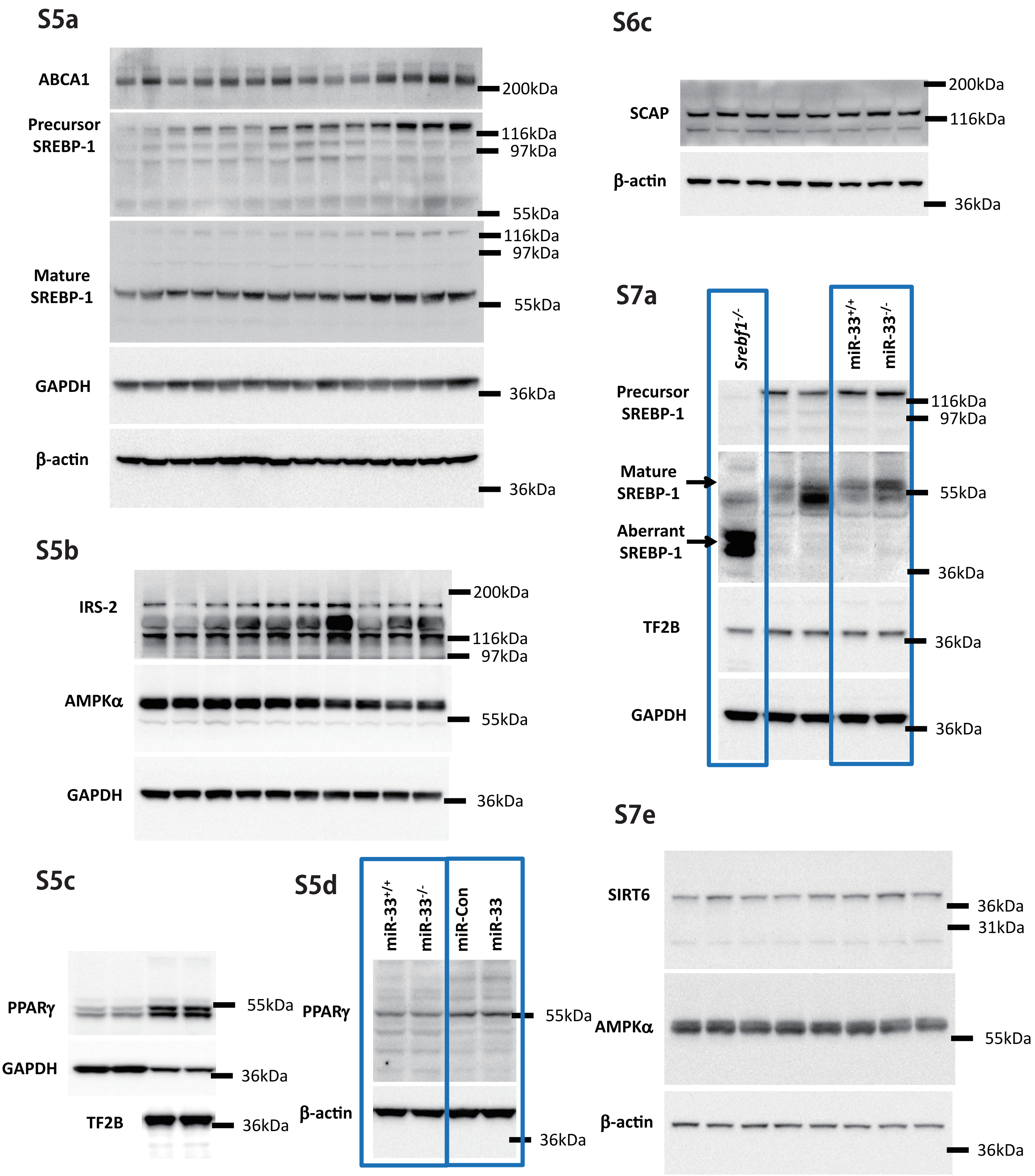


**Supplementary Figure S13. Scans of the original blots in Supplementary Figure S5a-d, S6c, S7a and S7e**

# Supplementary Table S1

Used gene-specific oligonucleotide primer sequences

| Gene | Species | Forward / Reverse |
|---|---|---|
| *SREBF1* | Human | 5'-AACAGTCCCACTGGTCGTAGAT-3'/ 5'-TGTTGCAGAAAGCGAATGTAGT-3' |
| *SREBF1a* | Human | 5'-ATGGACGAGCCACCCTTC-3'/ 5'-GCCAGGGAAGTCACTGTCTTG-3' |
| *SREBF1c* | Human | 5'-GCGCCTTGACAGGTGAAGTC-3'/ 5'-GCCAGGGAAGTCACTGTCTTG-3' |
| *ABCA1* | Human | 5'-GTCCTCTTTCCCGCATTATCTGG-3'/ 5'-AGTTCCTGGAAGGTCTTGTTCAC-3' |
| *IRS2* | Human | 5'-AGAGGACCTACTCCCTGACCAC-3'/ 5'-GATCTCGATGTCTCCGTAGTCC-3' |
| *ACTB* | Human | 5'-AGGCACTCTTCCAGCCTTCC-3'/ 5'-GCACTGTGTTGGCGTACAGG-3' |
| *Srebf1* | Mouse | 5'-TAGAGCATATCCCCCAGGTG-3'/ 5'-GGTACGGGCCACAAGAAGTA-3' |
| *Srebf1a* | Mouse | 5'-GGCCGAGATGTGCGAACT-3'/ 5'-TTGTTGATGAGCTGGAGCATGT-3' |
| *Srebf1c* | Mouse | 5'-GGAGCCATGGATTGCACATT-3'/ 5'-GAAGTCACTGTCTTGGTTGTTG-3' |
| *Srebf2* | Mouse | 5'-GTGGAGCAGTCTCAACGTCA-3'/ 5'-TGGTAGGTCTCACCCAGGAG-3' |
| *Scd1* | Mouse | 5'-GCGATACACTCTGGTGCTCA-3'/ 5'-CCCAGGGAAACCAGGATATT-3' |
| *Acaca (Acc1)* | Mouse | 5'-AGAAACCCGAACAGTGGAACT-3'/ 5'-AGGTAGCCCTTCACGGTTAAA-3' |
| *Fasn* | Mouse | 5'-GGGTTCTAGCCAGCAGAGTCTA-3'/ 5'-TGAGATGTGGATACCACCAGAG-3' |
| *Thrsp (Spot14)* | Mouse | 5'-TGACAGTCATGGATCGGTACTC-3'/ 5'-TGGTCCACTTCTACACAGATGC-3' |
| *Irs2* | Mouse | 5'-ACAACCTATCGTGGCACCTC-3'/ 5'-CCATGAGACTTAGCCGCTTC-3' |
| *Hmgcr* | Mouse | 5'-CGTAACCCAAAGGGTCAAGA-3'/ 5'-GACCCAAGGAAACCTTAGCC-3' |
| *Ldlr* | Mouse | 5'-ATTGGGTTGATTCCAAACTCC-3'/ 5'-ATTCACATCTGAACCCGTGAG-3' |
| *Cidea* | Mouse | 5'-ATGATCTTGGAAAAGGGACAGA-3'/ 5'-GCCTGTATAGGTCGAAGGTGAC-3' |
| *Cideb* | Mouse | 5'-GCCTGCTAAGGTCAGTATCCAC-3'/ 5'-CAGTGTTAGCACTCCACGTAGC-3' |
| *Cidec* | Mouse | 5'-GACCCAACAGCTGGTGTCTAA-3'/ 5'-TTCAAGATGTCCTGGACCTTG-3' |
| *Pparg* | Mouse | 5'-CCCACCAACTTCGGAATCAG-3'/ 5'-TGCTGGAGAAATCAACTGTGGTA-3' |
| *Cd36* | Mouse | 5'-GGCCAAGCTATTGCGACAT-3'/ 5'-CAGATCCGAACACAGCGTAGA-3' |
| *Ap2* | Mouse | 5'-GTGATGCCTTTGTGGGAACCTGGAAG-3'/5'-TCATAAACTCTTGTGGAAGTCACGCC-3' |
| *Ucp2* | Mouse | 5'-CAGGTCACTGTGCCCTTACCAT-3'/ 5'-CACTACGTTCCAGGATCCCAAG-3' |
| *Vldlr* | Mouse | 5'-TGTAGTCGTGGCTATCAAATGG-3' / 5'-TCTCTCTAGGCCAATCTTCCTG-3' |
| *Mogat1* | Mouse | 5'-CCTGGCTTTACATCGTATCTCC-3'/ 5'-GCTCAGCACATGAGACAAACTC-3' |
| *Dgat1* | Mouse | 5'-CCAACCATCTGATCTGGCTTA-3'/ 5'-CAGCATTCCACCAATCTCTGT-3' |
| *Mlxipl (Chrebp)* | Mouse | 5'-CCTCACTTCACTGTGCCTCA-3'/ 5'-ACAGGGGTTGTTGTCTCTGG-3' |
| *Cpt1a* | Mouse | 5'-GATCTACAATTCCCCTCTGCTCT-3'/ 5'-TAGAGCCAGACCTTGAAGTAACG-3' |
| *Crot* | Mouse | 5'-TACTTTTACCACGGCCGAAC-3'/ 5'-GACGGTCAAATCCTTTTCCA-3' |
| *Prkaa1* | Mouse | 5'-AGAGGGCCGCAATAAAAGAT-3'/ 5'-TGTTGTACAGGCAGCTGAGG-3' |
| *Cyp4a10* | Mouse | 5'-ATTTATGGTCTCCACCACAACC-3'/ 5'-CACAATCACCTTCAGCTCACTC-3' |
| *Cyp4a14* | Mouse | 5'-CTCAGCTCACCTGTCACCTTC-3'/ 5'-ATCCTCCTGAGAATGGCAGAT-3' |
| *Ehhadh* | Mouse | 5'-GCCTTACTGGACCGTCCTTAC-3'/ 5'-ACCCAGTGGCTTGTCATACTG-3' |
| *Angptl4* | Mouse | 5'-CTTCTCTACTTGGGACCAAGACC-3'/ 5'-CGTGGGATAGAGTGGAAGTATTG-3' |
| *Abca1* | Mouse | 5'-AACAGTTTGTGGCCCTTTTG-3'/ 5'-AGTTCCAGGCTGGGGTACTT-3' |
| *Actb* | Mouse | 5'-GATCTGGCACCACACCTTCT-3'/ 5'-GGGGTGTTGAAGGTCTCAAA-3' |
| *Scap* | Mouse | 5'-GATGTGTTCCGGTCACCTCT-3' / 5'-TTGGTCCCTGAGCTGTCTCT-3' |
| *Sirt6* | Mouse | 5'-GGCTACGTGGATGAGGTGAT-3'/ 5'-GGCTCAGCCTTGAGTGCTAC-3' |

## Supplementary Table S2

Serum profile of NC-fed aged mice

| | miR-33$^{+/+}$ NC 50W (n=5) | miR-33$^{-/-}$ NC 50W (n=5) | |
|---|---|---|---|
| **AST (IU/L)** | 53.40±4.11 | 62.00±8.07 | |
| **ALT (IU/L)** | 36.20±3.65 | 63.20±16.83 | |
| **ALP (IU/L)** | 201.80±12.28 | 237.00±19.77 | |
| **T-CHO (mg/dL)** | 89.60±4.70 | 137.60±8.42 | ** |
| **TG (mg/dL)** | 25.00±1.52 | 18.80±1.66 | * |
| **NEFA (μEq/L)** | 807.8±72.94 | 976.2±53.30 | |
| **LDL-C (mg/dL)** | 5.40±0.40 | 11.2±1.28 | ** |
| **HDL-C (mg/dL)** | 55.80±3.01 | 66.20±2.35 | * |

Values are the means ± s.e.m. Statistical comparisons were made by Student's t-test (*p < 0.05, **p < 0.01).

## Supplementary Table S3

Pathway analysis using GenMAPP software. Up-regulated pathways in miR-33$^{-/-}$ liver

| Pathway | **miR-33$^{-/-}$/miR-33$^{+/+}$ > 2** | |
|---|---|---|
| | gene | Z Score |
| Mm_Fatty_acid_metabolism | 5 | 7.449 |
| Mm_gamma_Hexachlorocyclohexane_degradation | 4 | 6.88 |
| Mm_Tryptophan_metabolism | 5 | 6.748 |
| Mm_Complement_and_Coagulation_Cascades_KEGG_WP449_33690 | 3 | 3.73 |
| Mm_Striated_Muscle_Contraction_WP216_33380 | 2 | 3.166 |
| Mm_Ascorbate_and_aldarate_metabolism | 1 | 2.799 |
| Mm_beta_Alanine_metabolism | 1 | 2.799 |
| Mm_Propanoate_metabolism | 1 | 2.674 |
| Mm_Complement_Activation,_Classical_Pathway_WP200_32694 | 1 | 2.457 |
| Mm_Endochondral_Ossification_WP1270_32869 | 2 | 2.35 |
| Mm_Glutathione_metabolism | 1 | 2.274 |
| Mm_Eicosanoid_Synthesis_WP318_35469 | 1 | 2.274 |
| Mm_Statin_Pathway(PharmGKB)_WP1_35542 | 1 | 2.193 |
| Mm_selenium_WP1272_34380 | 1 | 2.193 |
| Mm_Folic_Acid_Network_WP1273_34393 | 1 | 2.193 |

## Supplementary Table S4

Serum profile of mice with indicated genotype

| | **miR-33$^{+/+}$ *Srebf1*$^{+/+}$** | **miR-33$^{+/+}$ *Srebf1*$^{+/-}$** | **miR-33$^{-/-}$ *Srebf1*$^{+/+}$** | **miR-33$^{-/-}$ *Srebf1*$^{+/-}$** |
|---|---|---|---|---|
| **AST (IU/L)** | 60.2±3.94 | 66.17±4.09 | 101.67±33.60 | 53.50±3.10 |
| **ALT (IU/L)** | 22.67±2.53 | 37.67±4.76 | 73.33±45.79 | 25.50±5.38 |
| **ALP (IU/L)** | 144.83±8.77 | 144.33±5.06 | 188.33±10.93* | 144.83±12.93 |
| **T-CHO (mg/dL)** | 140.33±15.88 | 141.83±16.32 | 164.00±20.30 | 167.83±8.74 |
| **TG (mg/dL)** | 18.67±4.89 | 14.00±1.84 | 17.83±1.74 | 15.00±2.18 |
| **NEFA (μEq/L)** | 505.33±60.80 | 473.83±45.38 | 480.33±65.29 | 421.67±36.62 |
| **LDL-C (mg/dL)** | 12.83±2.80 | 10.00±1.37 | 13.83±2.83 | 13.67±1.48 |
| **HDL-C (mg/dL)** | 68.17±5.36 | 70.00±5.54 | 76.50±7.12 | 73.00±2.07 |

Values are the means ± s.e.m. Statistical comparisons were made by one-way analysis of valiance test (n= 6 each, *p < 0.05).
